# Supplementary material for: Genetic controllers for enhancing the evolutionary longevity of synthetic gene circuits in bacteria
Source: Nat Commun. 2025 Sep 29;16:8590. doi: 10.1038/s41467-025-63627-4 (PMC12479964; doi:10.1038/s41467-025-63627-4)
Supplement: Supplementary file 1 — Supplementary Information [file 41467_2025_63627_MOESM1_ESM.pdf]

SUPPLEMENTARY INFORMATION

Engineering genetic controllers to improve the evolutionary longevity of synthetic gene circuits in bacteria

Daniel P. Byrom[1] **and Alexander P.S. Darlington**[1,*]

[1]Warwick Integrative Synthetic Biology Centre, School of Engineering, University of Warwick, Coventry, CV4 7AL, UK

[*] correspondence to a.darlington.1@warwick.ac.uk

Supplementary Figures

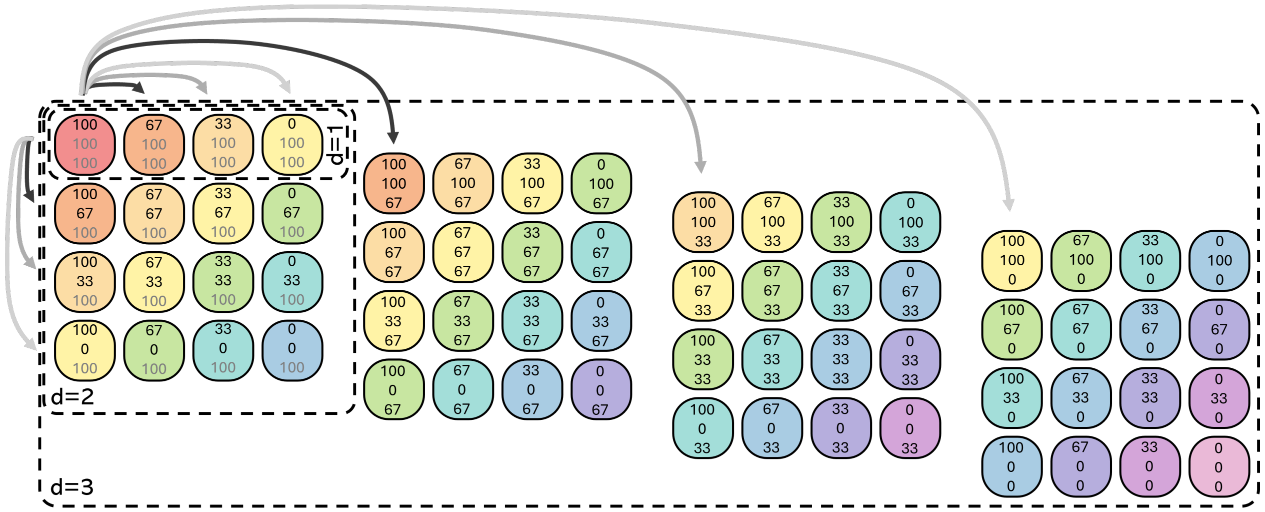

Supplementary Figure 1: A visual depiction of the mutation scheme used throughout the paper with the number of mutating dimensions $d = 1, 2, 3$. Coloured squares represent distinct mutation states with different levels of function. Numbers inside the squares show the percentage function of each 'mutatable' parameter, with 100 representing function at the designed level and 0 representing no function at all. Arrows signify possible transitions between mutation states, with lighter arrows showing mutations which occur less often. Only arrows from the fully functional state are displayed, and all such arrows are shown.

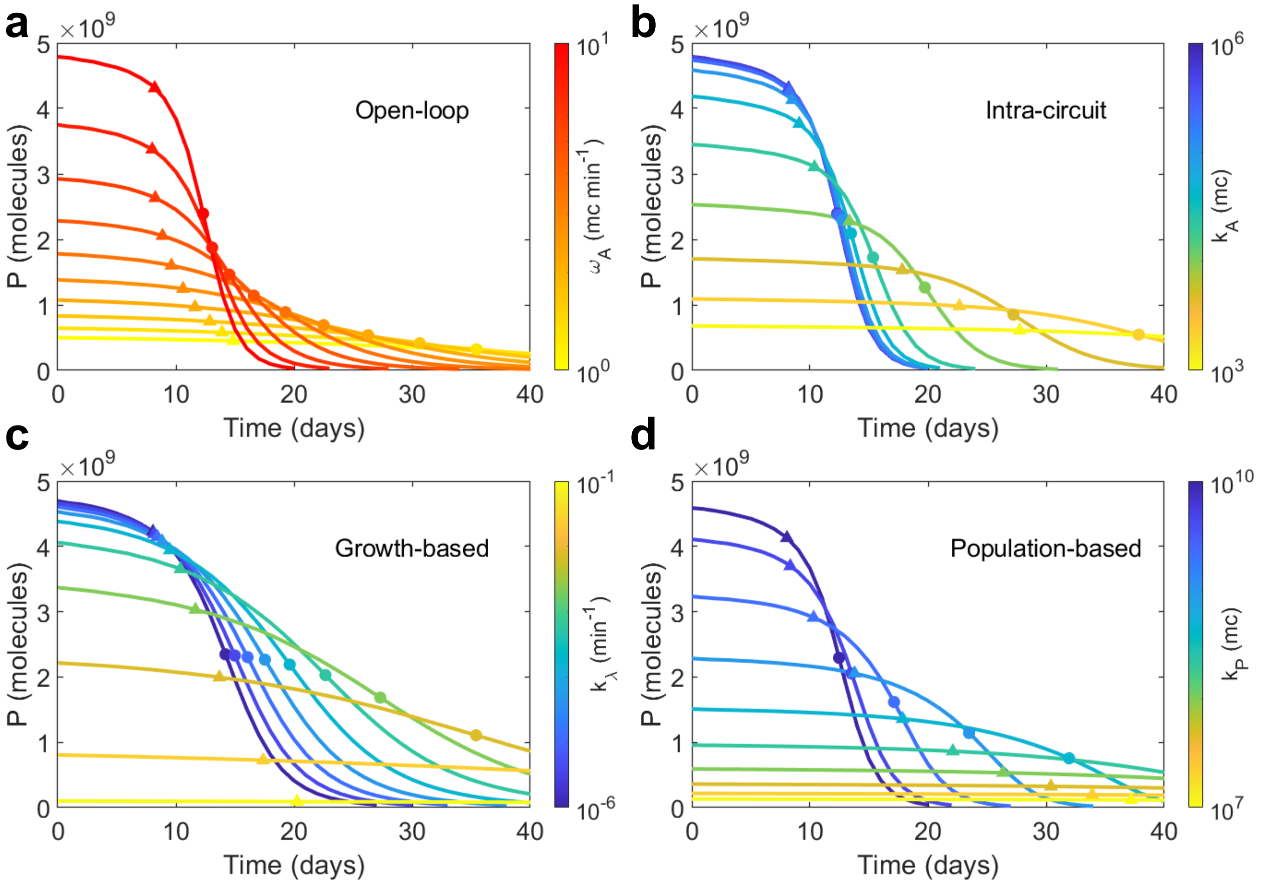

Supplementary Figure 2: Longevity can be increased at the expense of output. Plots show population-wide protein output P over time for different systems. Triangles indicate points where output falls to 90% of its original value P_0 and circles indicate points where output falls to 50% of P_0. (a) An open-loop system with maximal transcription rate ω_A varying between 1 and 10 mc min^{-1}. (b-d) Controller designs of varying strength (k_A, k_λ, k_P) applied to a fixed process with $\omega_A = 10$ mc min^{-1}. Lighter colours represent stronger control. (b) Intra-circuit control. (c) Growth-based control. (d) Population-based control.

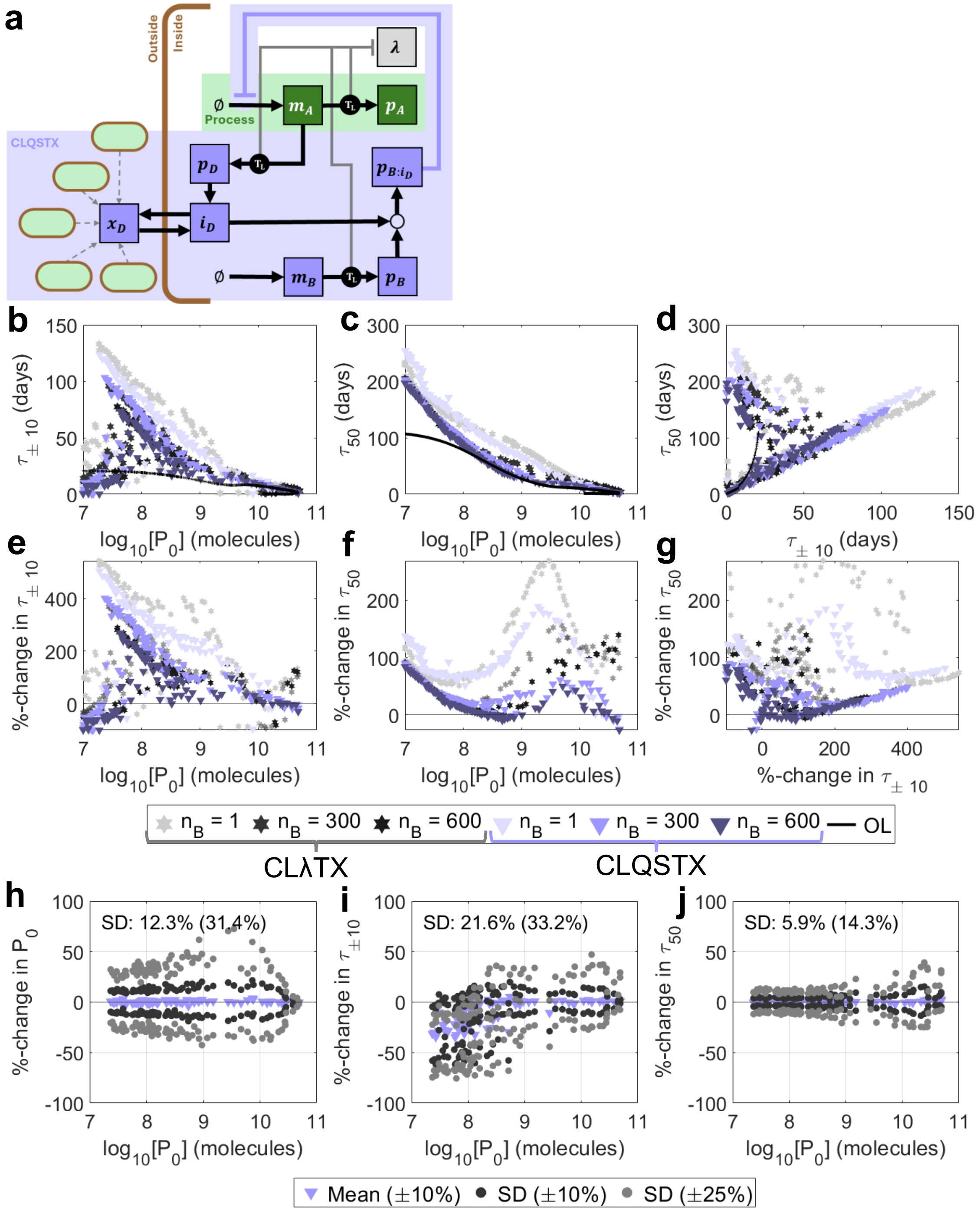

Supplementary Figure 3: Considering CLQSTX and comparing its performance against CLλTX. (a) A schematic describing CLQSTX. Synthetic enzyme p_D is produced from the same gene as the process p_A. This catalyses the production of internal metabolite i_D, which can be externalised to form the population-wide variable x_D and vice versa. A separate controller protein p_B is produced constitutively and binds with i_D to form a functional transcription factor $[p_{B:i_D}]$, which inhibits the production of process mRNA m_A. See key in Fig. 2a for symbol meanings. (b-g) Optimal performance for both CLλTX (grey) and CLQSTX (lilac) for controller protein length $n_B = 1, 300, 600$ aa. (b) $\tau_{\pm 10}$ vs initial output P_0, (c) τ_{50} vs initial output P_0, (d) τ_{50} vs $\tau_{\pm 10}$, (e) %-change in $\tau_{\pm 10}$ over open-loop vs initial output P_0, (f) %-change in τ_{50} over open-loop vs initial output P_0, (g) %-change in $\tau_{\pm 10}$ over open-loop vs %-change in τ_{50} over open-loop. (h-j) Robustness analysis. For each of the 100 optimal controllers for $n_B = 300$ aa, 100 further controllers were generated by varying parameters by up to ±10% (dark grey) and ±25% (light grey). The percentage changes in three output metrics were calculated versus the original optimal systems: (h) P_0, (i) $\tau_{\pm 10}$ and (j) τ_{50}. Plots show the means (for ±10%) and standard deviations (for both ±10% and ±25%) of the percentage changes for each optimal controller. Percentages marked on the plots indicate the standard deviations across the entire Pareto front when parameters were varied by ±10% (±25%). Only original controllers where $\tau_{\pm 10} = \tau_{90}$ are considered.

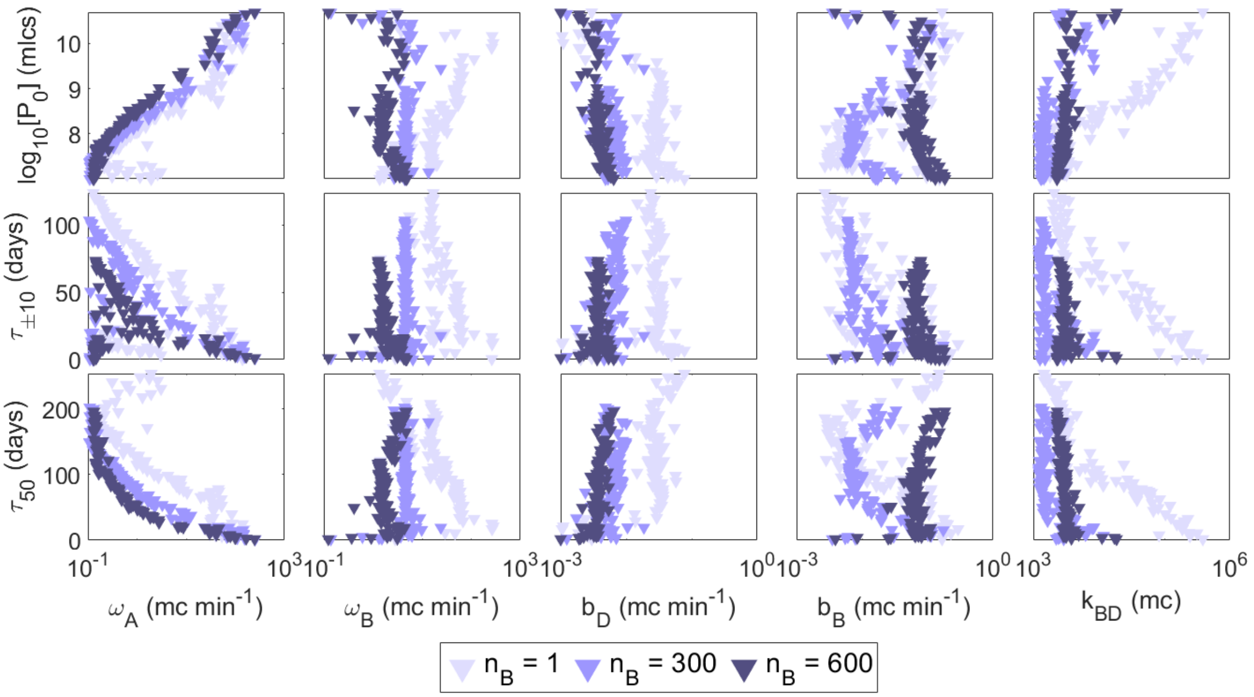

Supplementary Figure 4: Optimal parameter choices for population-based control designs. We performed multi-objective optimisations on CLQSTX for three controller sizes ($n_B = 1, 300, 600$ aa) to simultaneously maximise P_0, $\tau_{\pm 10}$ and τ_{50}. These three objectives are plotted against the optimal parameter choices for each system (ω_A, ω_B, b_D, b_B and k_{BD}), corresponding to outputs in Fig. S3.

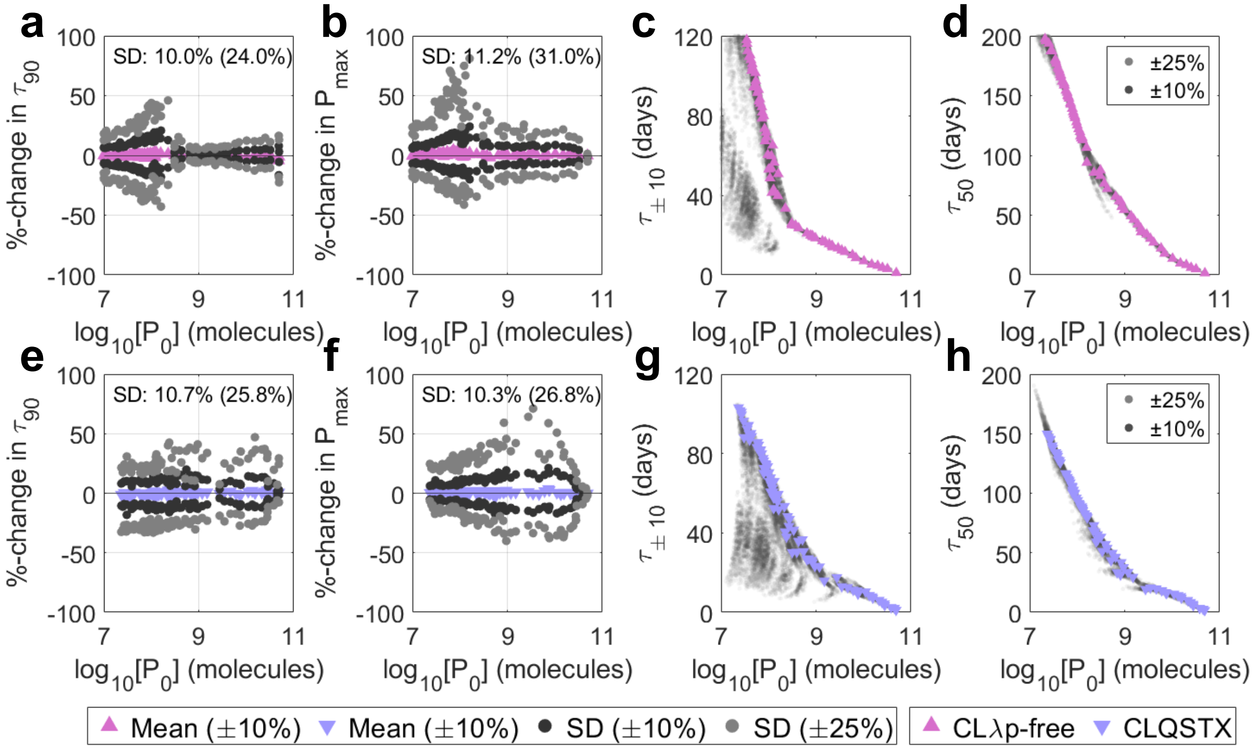

Supplementary Figure 5: Robustness analysis for (a-d) CLλprotein-free (pink) and (e-h) CLQSTX (lilac). 100 optimal controllers for each system were generated by multi-objective optimisation with $n_B = 300$ aa. For each of these, 100 further controllers were generated by varying parameters by up to ±10% (dark grey) and ±25% (light grey). (a,b,e,f) The percentage changes in two output metrics were calculated versus the original optimal systems: (a,e) τ_{90} and (b,f) P_{max}. Plots show the means (for ±10%) and standard deviations (for both ±10% and ±25%) of the percentage changes for each optimal controller. Percentages marked on the plots indicate the standard deviations across the entire Pareto front when parameters were varied by ±10% (±25%). (c,d,g,h) The impact of parameter variation on the Pareto front. Coloured markers indicate the original front which simultaneously maximises P_0, $\tau_{\pm 10}$ and τ_{50}. Grey markers indicate systems with varied parameters. Plots show (c,g) $\tau_{\pm 10}$ and (d,h) τ_{50} against initial output P_0. Only original controllers where $\tau_{\pm 10} = \tau_{90}$ are considered.

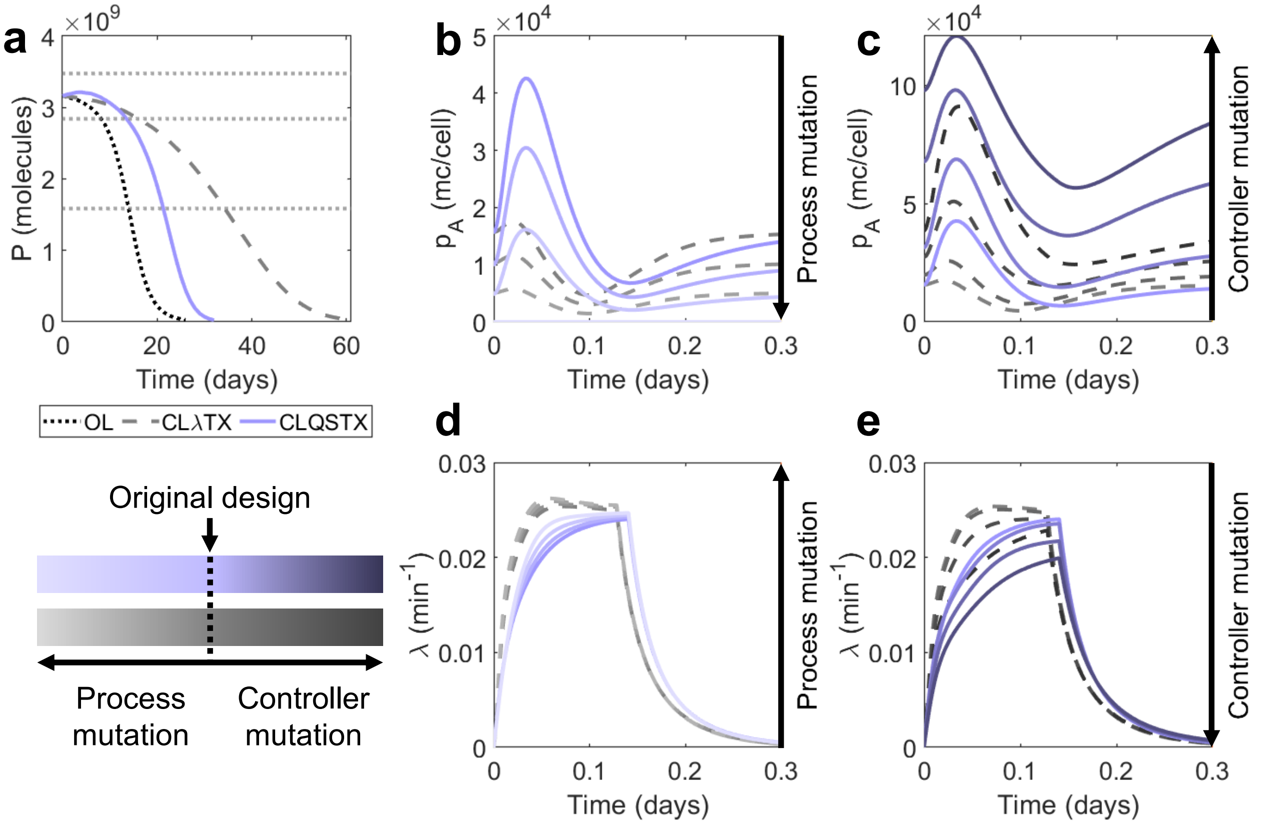

Supplementary Figure 6: Why does CLλTX outperform CLQSTX? (a) Time-series output for representative optimal controllers with $n_B = 300$ aa. (Open-loop black dotted line.) (b) Protein output per cell p_A over time for the first day of simulation according to mutation state, considering only states with mutated processes and fully functional controllers. (c) Protein output per cell p_A over time for the first day of simulation, considering only states with mutated controllers and fully functional processes. (d) Growth rate λ over time for the first day of simulation according to mutation state, considering only states with mutated processes. (e) Growth rate λ over time for the first day of simulation, considering only states with mutated controllers.

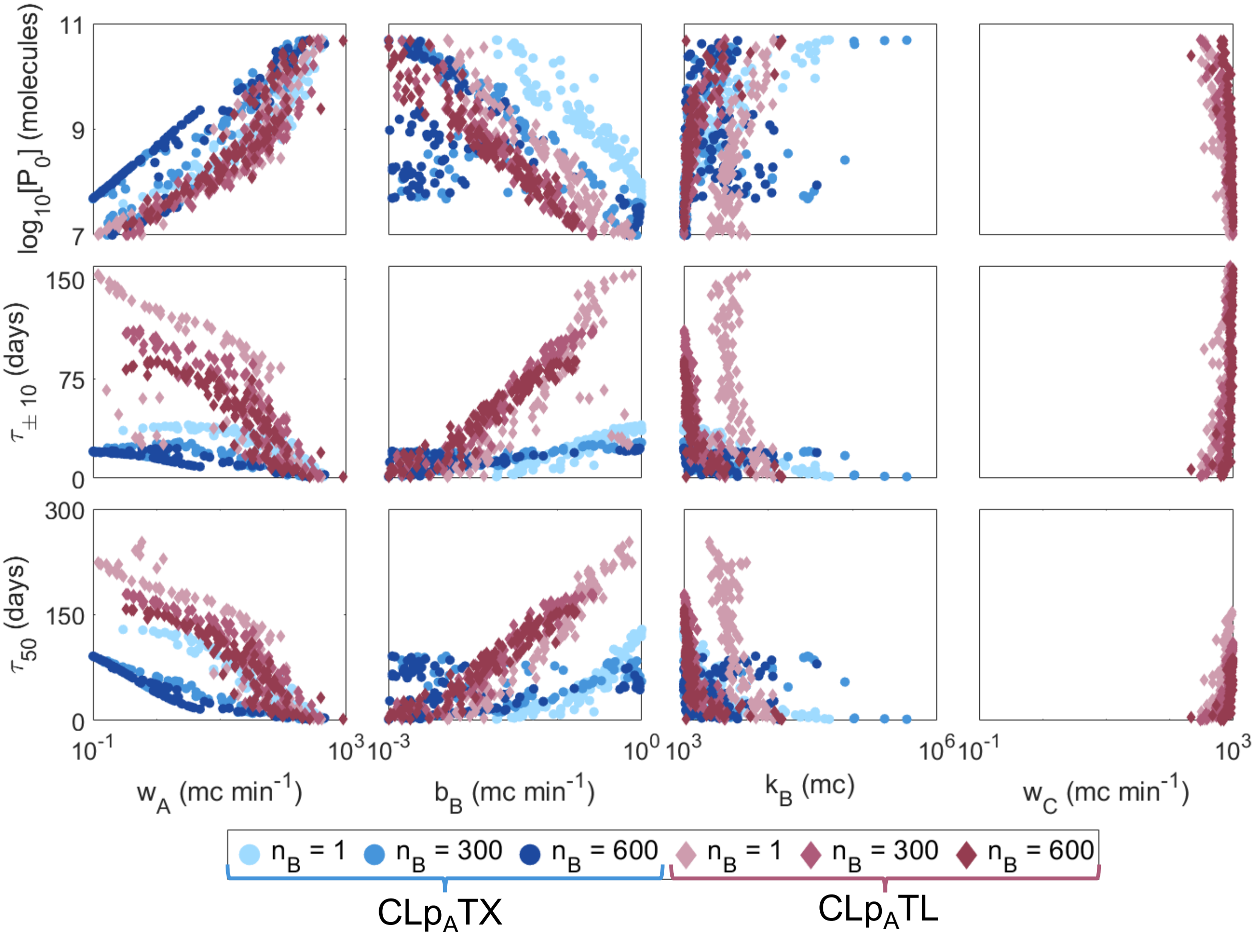

Supplementary Figure 7: Optimal parameter choices for protein-based control designs. We performed multi-objective optimisations on CLp_ATX and CLp_ATL for three controller sizes ($n_B = 1, 300, 600$ aa) to simultaneously maximise P_0, $\tau_{\pm 10}$ and τ_{50}. These three objectives are plotted against the optimal parameter choices for each system (ω_A, b_B, k_B and ω_C), corresponding to outputs in Fig. 4. (Note ω_C is not included in the model for CLp_ATX.)

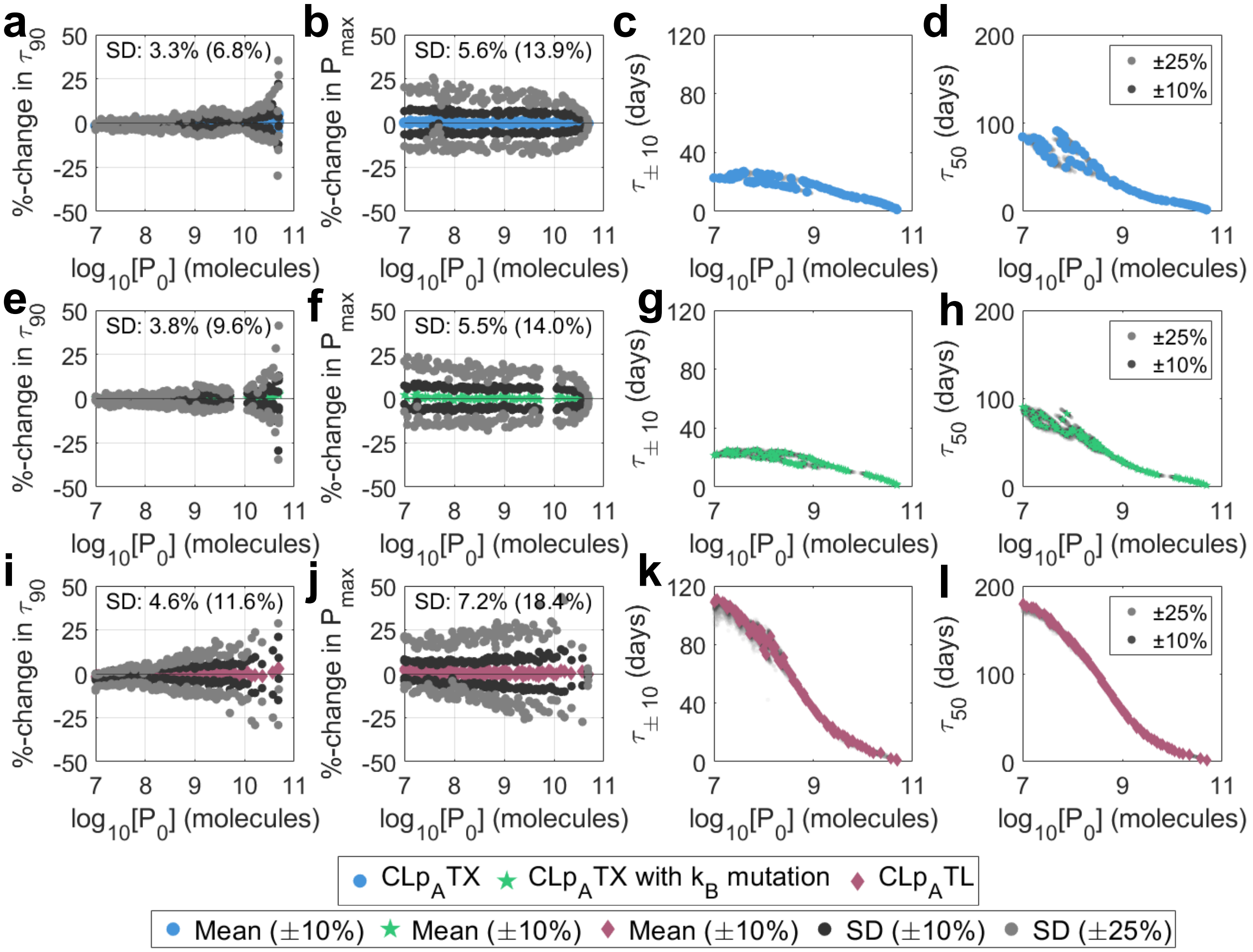

Supplementary Figure 8: Robustness analysis for (a-d) CLp_ATX (blue), (e-h) CLp_ATX with mutation of the k_B parameter (green), (i-l) CLp_ATL (red). 100 optimal controllers for each system were generated by multi-objective optimisation with $n_B = 300$ aa. For each of these, 100 further controllers were generated by varying parameters by up to ±10% (dark grey) and ±25% (light grey). (a,b,e,f,i,j) The percentage changes in two output metrics were calculated versus the original optimal systems: (a,e,i) τ_{90} and (b,f,j) P_{max}. Plots show the means (for ±10%) and standard deviations (for both ±10% and ±25%) of the percentage changes for each optimal controller. Percentages marked on the plots indicate the standard deviations across the entire Pareto front when parameters were varied by ±10% (±25%). (c,d,g,h,k,l) The impact of parameter variation on the Pareto front. Coloured markers indicate the original front which simultaneously maximises P_0, $\tau_{\pm 10}$ and τ_{50}. Grey markers indicate systems with varied parameters. Plots show (c,g,k) $\tau_{\pm 10}$ and (d,h,l) τ_{50} against initial output P_0. Only original controllers where $\tau_{\pm 10} = \tau_{90}$ are considered.

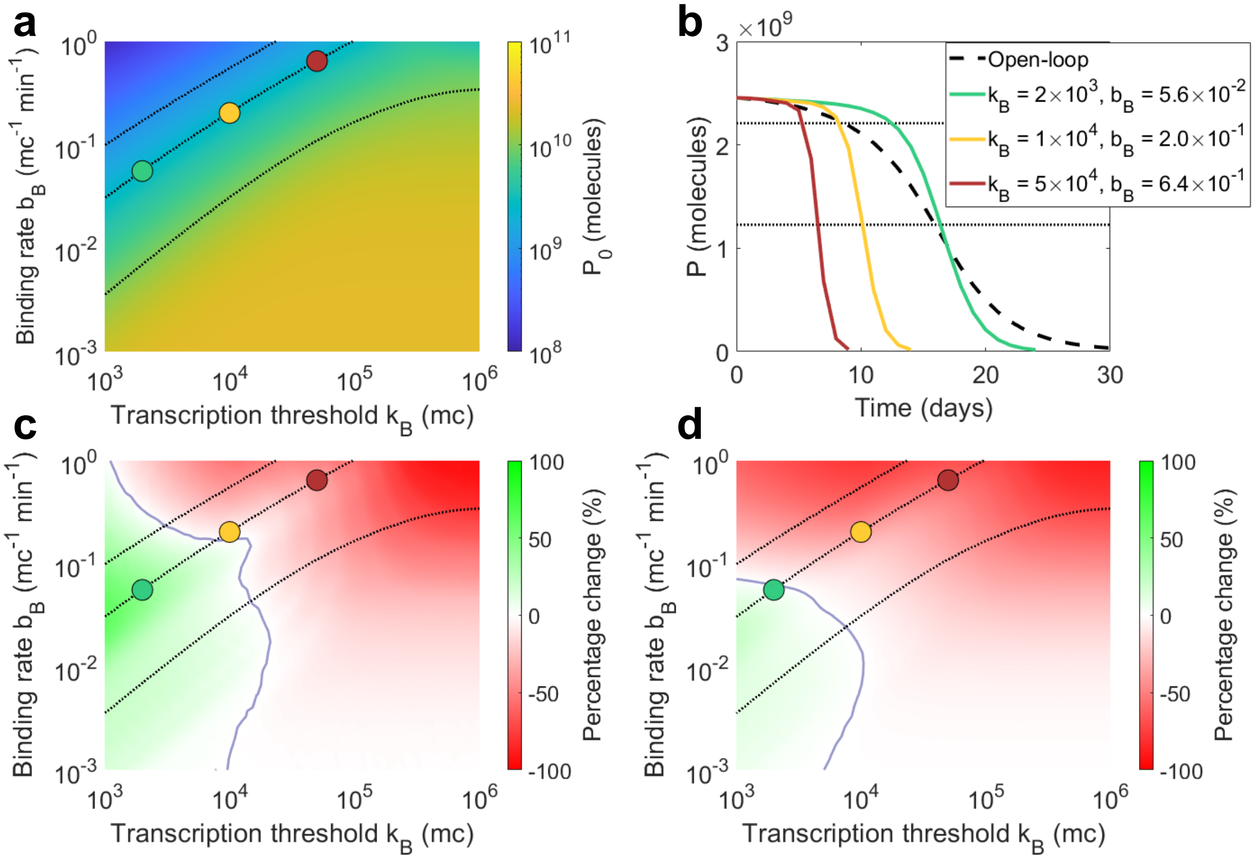

Supplementary Figure 9: Designing an intra-circuit controller CLp_ATX for a nominal process with $\omega_A = 50$ mc min^{-1}. (a) Heatmap of initial protein output P_0 for different parameter combinations as ribosome binding rate b_B and control strength k_B are varied. Contours plotted for 1×10^9, 2.5×10^9, 1×10^{10} mc. Markers correspond to parameterizations in (b). (b) Time series protein output for three different controller parameterizations that each produce the same initial output. The dotted line marks the open-loop system. The yellow line describes a basic controller with $k_B = 1 \times 10^4$ mc, $b_B = 10^{-1}$mc^{-1}min^{-1}. The red line describes a weaker binding, highly expressed controller (high k_B, high b_B). The green line describes a tighter binding, less expressed controller (low k_B, low b_B). (c,d) Heatmap showing the percentage change in (c) $\tau_{\pm10}$ and (d) τ_{50} for different parameter combinations. Green areas represent an improvement with feedback, red areas represent a worsening, and white areas represent little change. The blue contour marks the boundary where there is no change. Black contours are analogous to those in (a). Markers correspond to parameterizations in (b).

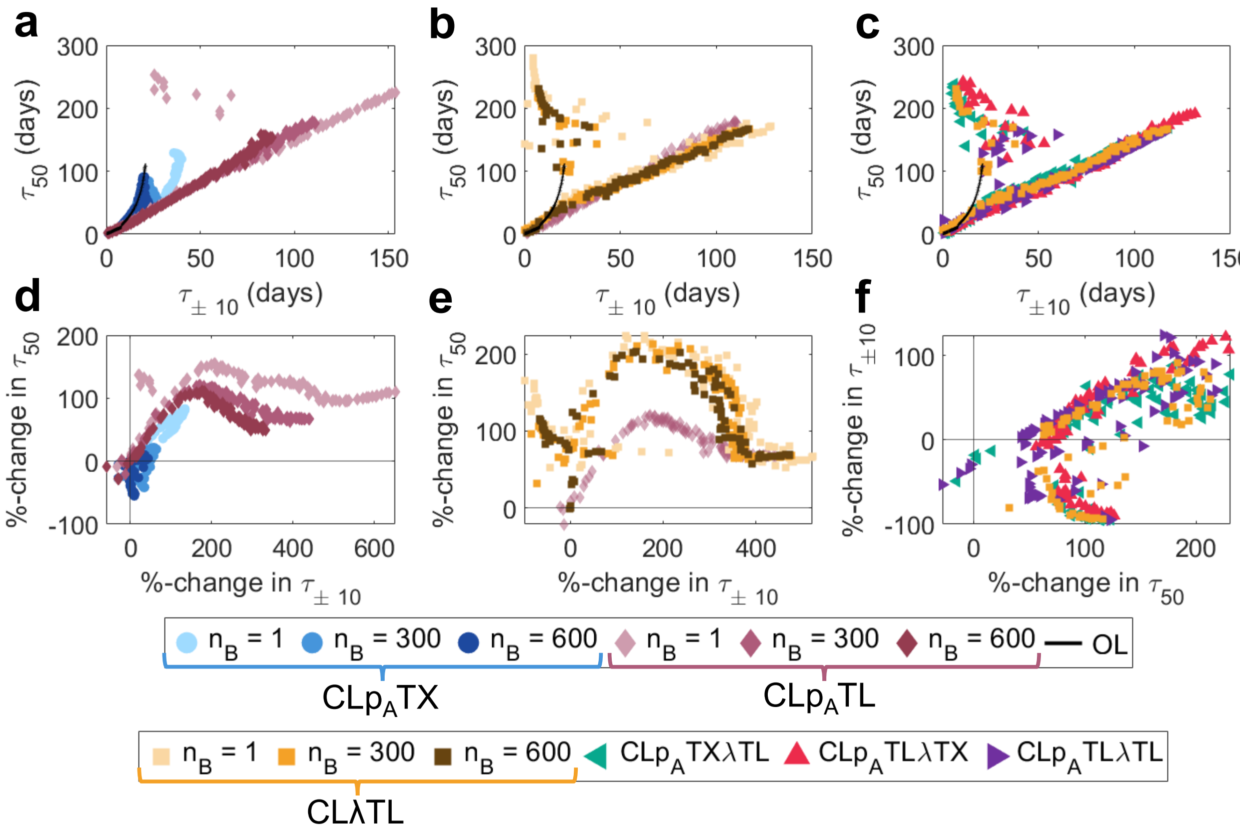

Supplementary Figure 10: The relationship between $\tau_{\pm 10}$ and τ_{50} for various controllers. Optimisations were performed to simultaneously maximise P_0, $\tau_{\pm 10}$ and τ_{50} for the following controllers: (a,d) CLp_ATX (blue) and CLp_ATL (red) for $n_B = 1, 300, 600$ aa, (b,e) CLλTL (orange) for $n_B = 1, 300, 600$ aa and CLp_ATL (red) for $n_B = 300$ aa, (c,f) CLλTL (orange), CLp_ATXλTL (teal), CLp_ATLλTX (crimson) and CLp_ATLλTL (purple) for $n_B = 300$ aa. (a-c) τ_{50} against $\tau_{\pm 10}$, (d-f) percentage change in τ_{50} vs open-loop against percentage change in $\tau_{\pm 10}$ vs open-loop.

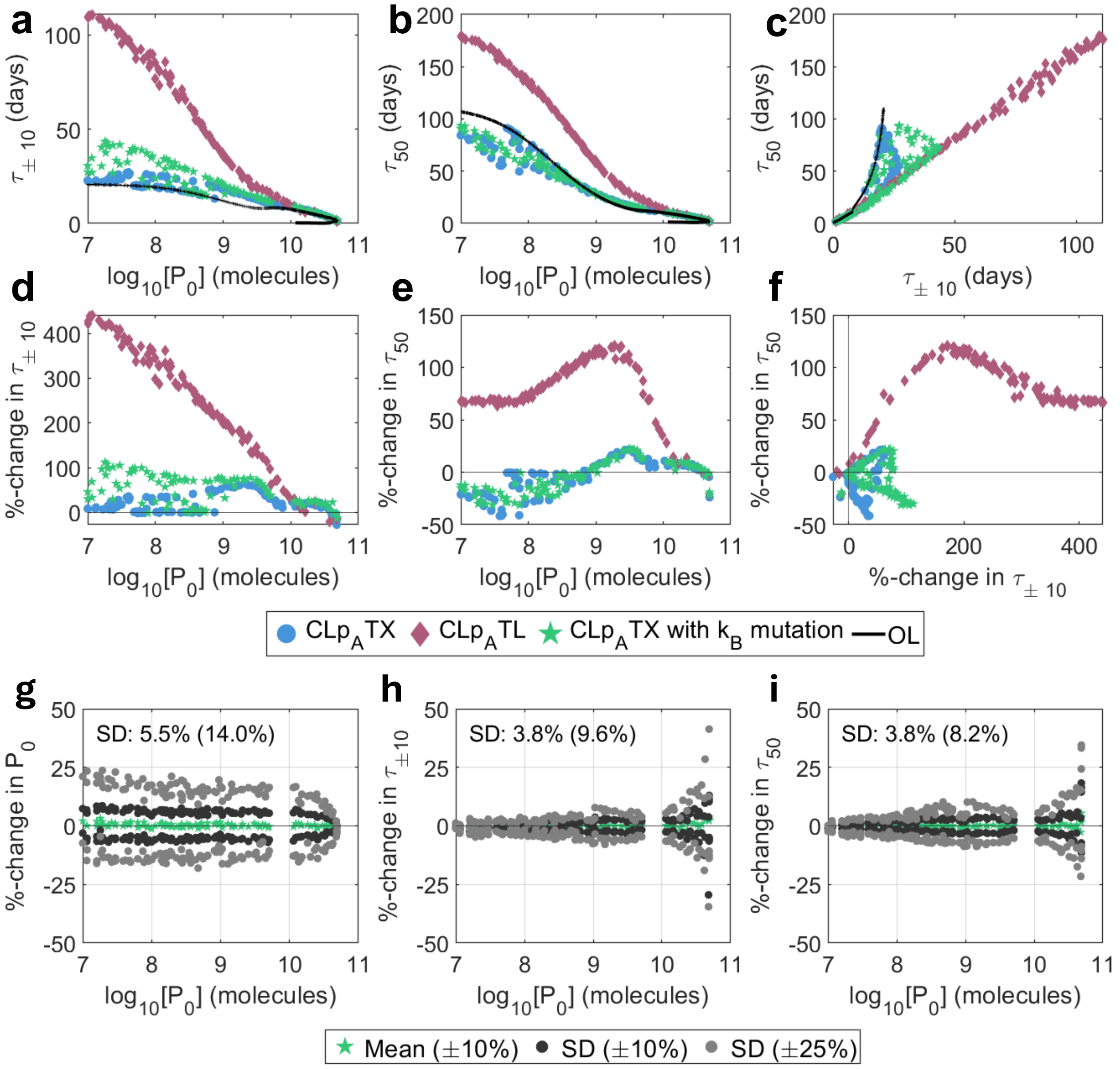

Supplementary Figure 11: Considering mutation of both maximal transcription rate ω_A and controller strength k_B for CLp_ATX. (a-f) Optimal performance. In each plot, we show CLp_ATX (one dimension of mutation, blue), CLp_ATL (two dimensions of mutation, red) and CLp_ATX with additional mutation of the parameter k_B, scaled so that $k_B = 10^6$ mc represents a non-functional controller (two dimensions of mutation, green). $n_B = 300$ aa. (a) $\tau_{\pm 10}$ vs initial output P_0, (b) τ_{50} vs initial output P_0, (c) τ_{50} vs $\tau_{\pm 10}$, (d) %-change in $\tau_{\pm 10}$ over open-loop vs initial output P_0, (e) %-change in τ_{50} over open-loop vs initial output P_0, (f) %-change in $\tau_{\pm 10}$ over open-loop vs %-change in τ_{50} over open-loop. (g-i) Robustness analysis. For each of the 100 optimal controllers, 100 further controllers were generated by varying parameters by up to ±10% (dark grey) and ±25% (light grey). The percentage changes in three output metrics were calculated versus the original optimal systems: (g) P_0, (h) $\tau_{\pm 10}$ and (i) τ_{50}. Plots show the means (for ±10%) and standard deviations (for both ±10% and ±25%) of the percentage changes for each optimal controller. Percentages marked on the plots indicate the standard deviations across the entire Pareto front when parameters were varied by ±10% (±25%).

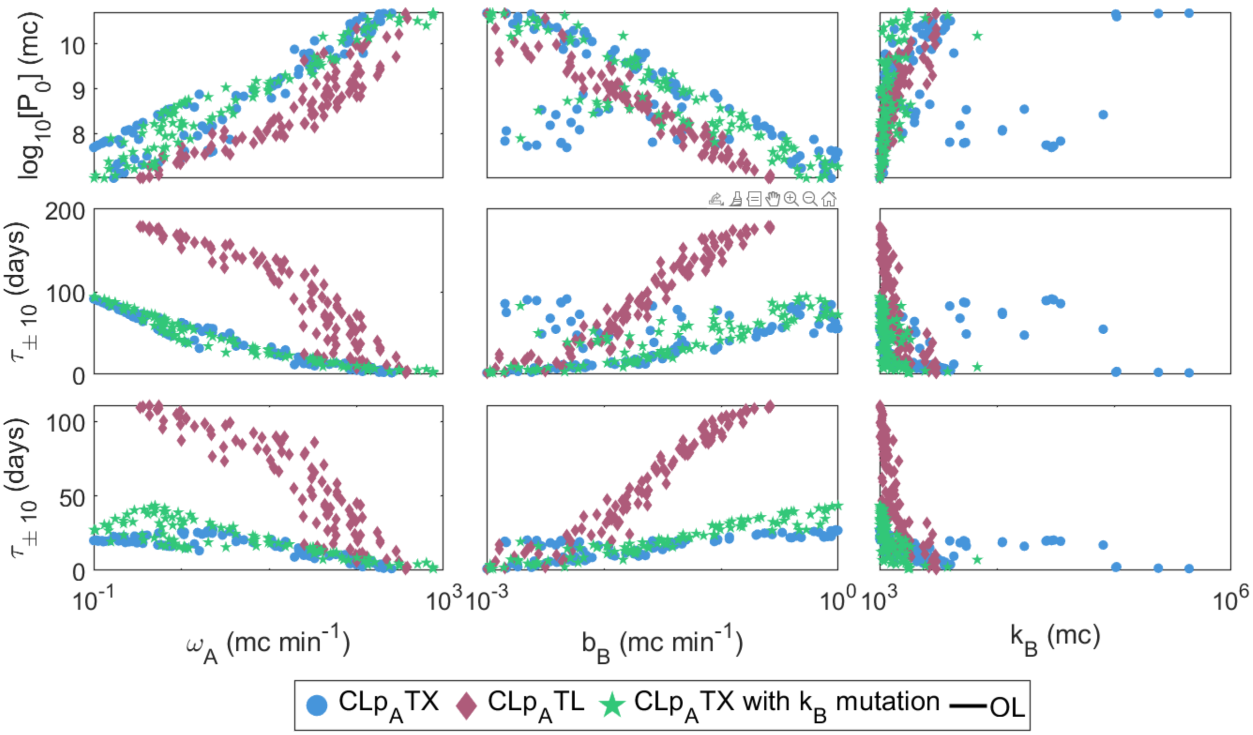

Supplementary Figure 12: Optimal parameters when both maximal transcription rate ω_A and controller strength k_B can mutate for CLp_ATX. We performed multi-objective optimisations to simultaneously maximise P_0, $\tau_{\pm 10}$ and τ_{50}. These three objectives are plotted against the optimal parameter choices for each system (ω_A, b_B and k_B), corresponding to outputs in Fig. S11. We show CLp_ATX (one dimension of mutation, blue), CLp_ATL (two dimensions of mutation, red) and CLp_ATX with additional mutation of the parameter k_B, scaled so that $k_B = 10^6$ mc represents a non-functional controller (two dimensions of mutation, green).

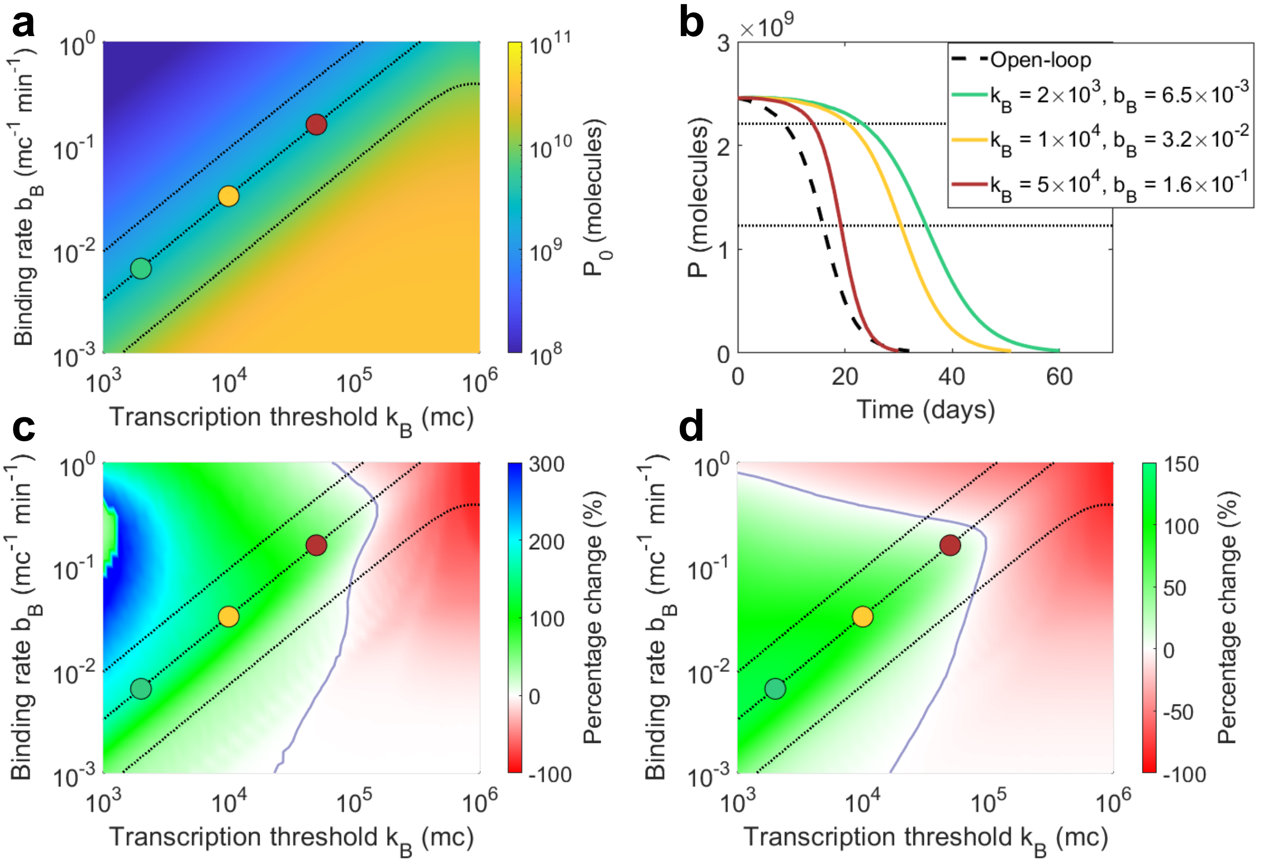

Supplementary Figure 13: Designing a post-transcriptional intra-circuit controller CLp_ATL for a nominal process with $\omega_A = 50$ mc min^{-1}. Maximal sRNA transcription rate ω_C fixed at its maximum of 1000 mc min^{-1}. (a) Heatmap of initial protein output P_0 for different parameter combinations as ribosome binding rate b_B and control strength k_B are varied. Contours plotted for 1×10^9, 2.5×10^9, 1×10^{10} mc. Markers correspond to parameterizations in (b). (b) Time series protein output for three different controller parameterizations that each produce the same initial output. The dotted line marks the open-loop system. The yellow line describes a basic controller with $k_B = 1 \times 10^4$ mc, $b_B = 3.2 \times 10^{-2}$mc^{-1}min^{-1}. The red line describes a weaker binding, highly expressed controller (high k_B, high b_B). The green line describes a tighter binding, less expressed controller (low k_B, low b_B). (c,d) Heatmap showing the percentage change in (c) $\tau_{\pm 10}$ and (d) τ_{50} for different parameter combinations. Green and blue areas represent an improvement with feedback, red areas represent a worsening, and white areas represent little change. The blue contour marks the boundary where there is no change. Black contours are analogous to those in (a). Markers correspond to parameterizations in (b).

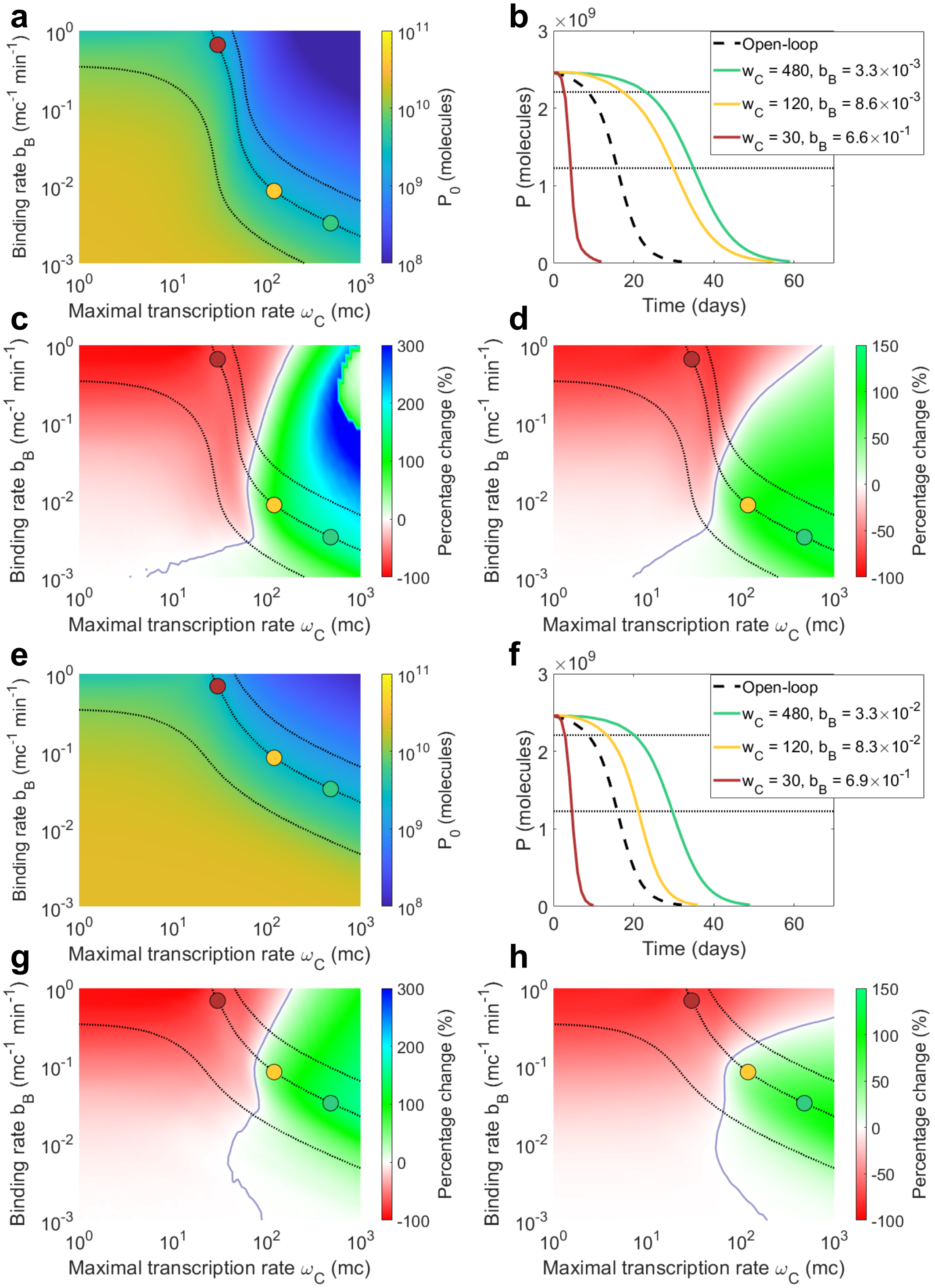

Supplementary Figure 14: Designing a post-transcriptional intra-circuit controller CLp_ATL for a nominal process with $\omega_A = 50$ mc min^{-1}. Controller threshold parameter k_B fixed at (a-d) 10^3 mc^{-1} min^{-1} and (e-h) 10^4 mc^{-1} min^{-1}. (a,e) Heatmap of initial protein output P_0 for different parameter combinations as maximal sRNA transcription rate ω_C and control strength k_B are varied. Contours plotted for 1×10^9, 2.5×10^9, 1×10^{10} mc. Markers correspond to parameterizations in (b,f). (b,f) Time series protein output for three different controller parameterizations that each produce the same initial output. The dotted line marks the open-loop system. The red line describes a controller with less abundant sRNA and more abundant transcription factors (low ω_C, high b_B). The green line describes a controller with more abundant sRNA and less abundant transcription factors (high ω_C, low b_B). (c,d,g,h) Heatmap showing the percentage change in (c,g) $\tau_{\pm 10}$ and (d,h) τ_{50} for different parameter combinations. Green and blue areas represent an improvement with feedback, red areas represent a worsening, and white areas represent little change. The blue contour marks the boundary where there is no change. Black contours are analogous to those in (a). Markers correspond to parameterizations in (b).

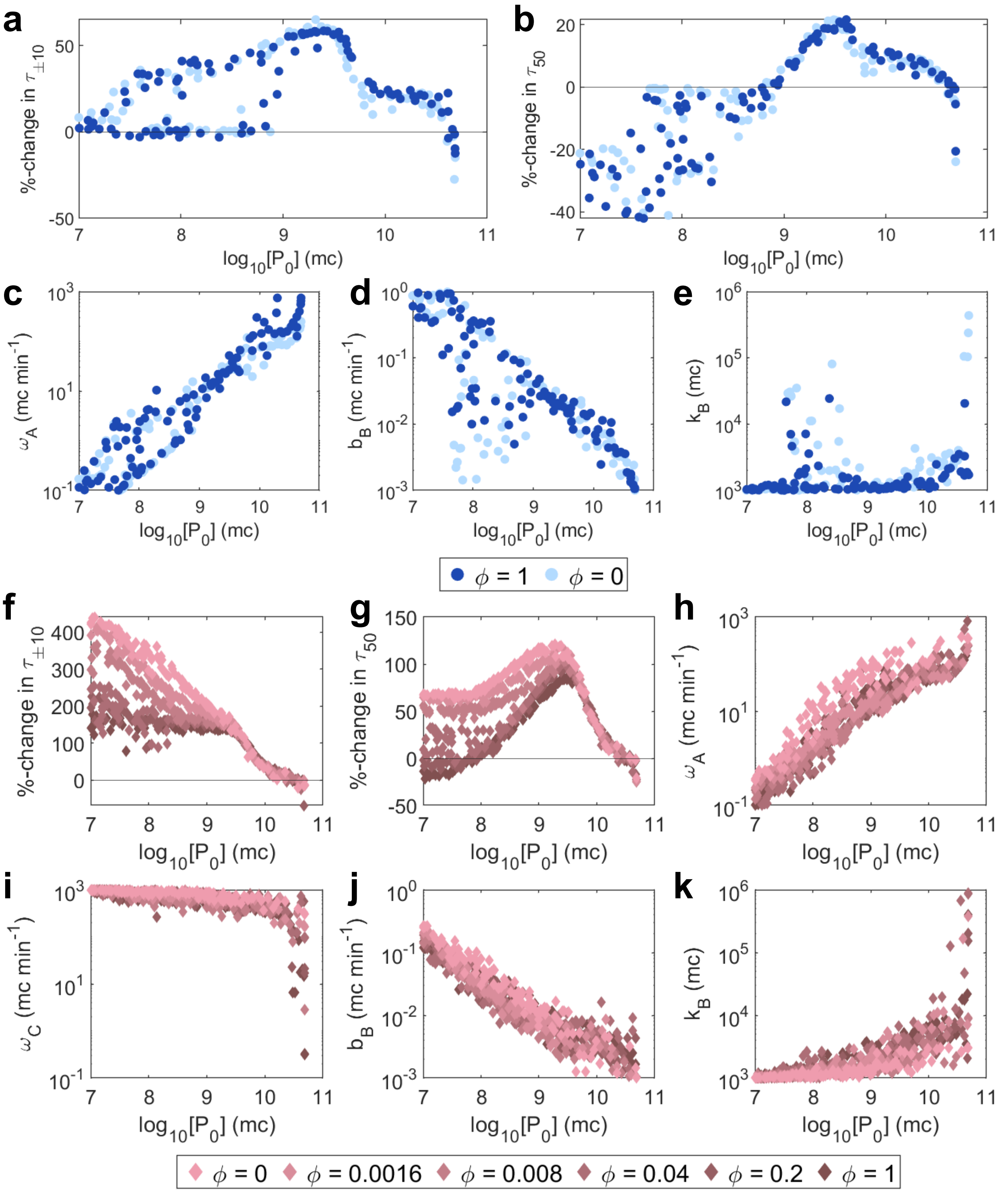

Supplementary Figure 15: Evaluating the impact of transcriptional burden on (a-e) CLp_ATX (blue) and (f-k) CLp_ATL (red). For CLp_ATX, we consider systems with $\phi_{T_X} = 1$ and $\phi_{T_X} = 0$. For CLp_ATL, we also consider systems with $\phi_{T_X} = 0.2, 0.04, 0.008, 0.0016$. Here, ϕ_{T_X} represents the transcriptional cost of the production of synthetic RNA. We performed multi-objective optimisations simultaneously optimising P_0, $\tau_{\pm10}$ and τ_{50} and compared the outputs against open-loop systems of equivalent P_0 and ϕ_{T_X}. All plots have initial output P_0 on the x-axis. (a,f) $\tau_{\pm10}$, (b-g) τ_{50}, (c,h) ω_A, (d,j) k_B, (e,k) k_B, (i) ω_C.

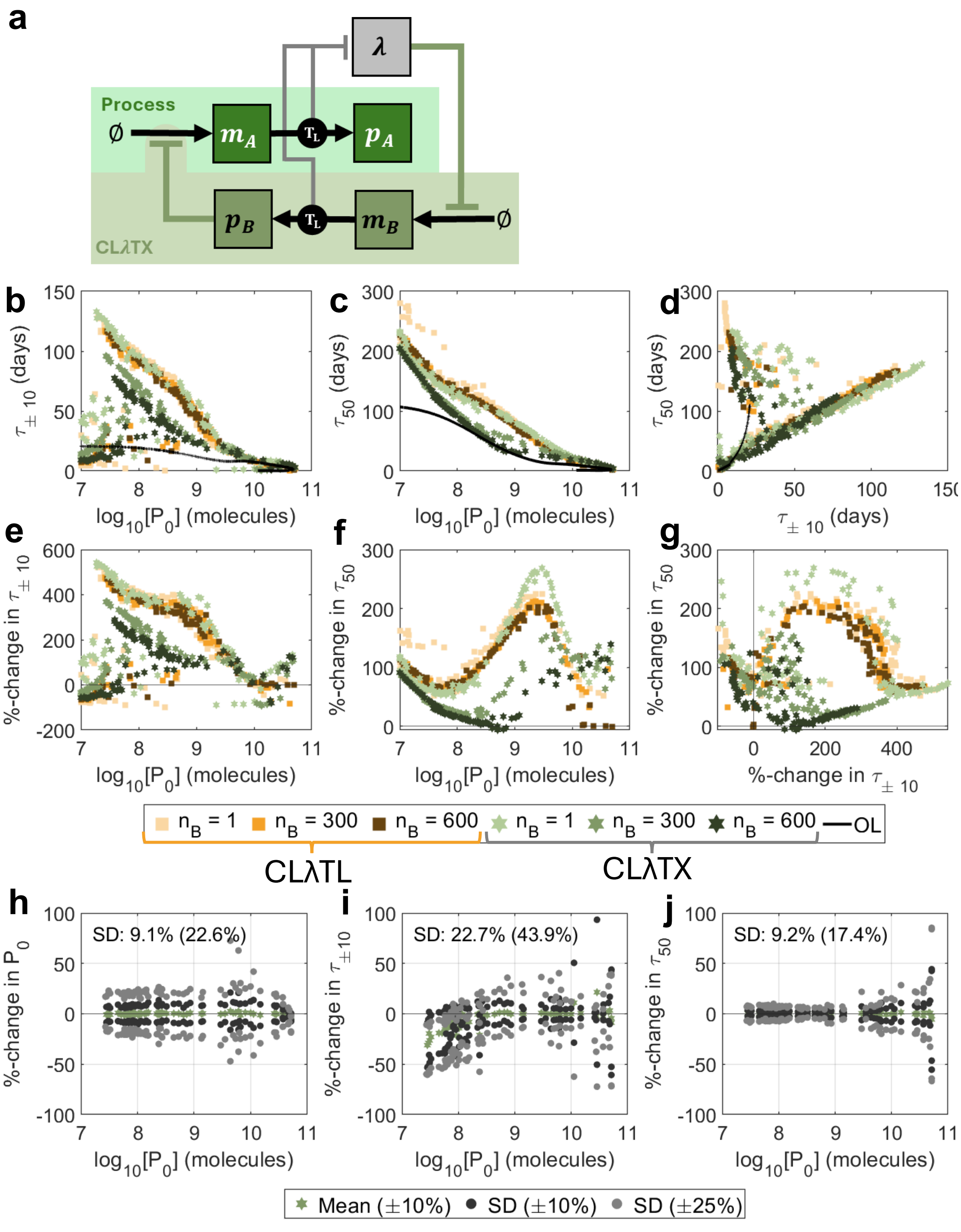

Supplementary Figure 16: Considering CLλTX and comparing its performance against CLλTL. (a) A schematic describing CLλTX. Controller protein p_B is produced from a growth-sensitive promoter and acts as a transcription factor to inhibit the production of process mRNA m_A. (b-g) Optimal performance for both CLλTL (orange) and CLλTX (olive) for controller protein length $n_B = 1, 300, 600$ aa. See key in Fig. 2a for symbol meanings. (b) $\tau_{\pm 10}$ vs initial output P_0, (c) τ_{50} vs initial output P_0, (d) τ_{50} vs $\tau_{\pm 10}$, (e) %-change in $\tau_{\pm 10}$ over open-loop vs initial output P_0, (f) %-change in τ_{50} over open-loop vs initial output P_0, (g) %-change in $\tau_{\pm 10}$ over open-loop vs %-change in τ_{50} over open-loop. (h-j) Robustness analysis. For each of the 100 optimal controllers for $n_B = 300$ aa, 100 further controllers were generated by varying parameters by up to $\pm 10\%$ (dark grey) and $\pm 25\%$ (light grey). The percentage changes in three output metrics were calculated versus the original optimal systems: (h) P_0, (i) $\tau_{\pm 10}$ and (j) τ_{50}. Plots show the means (for $\pm 10\%$) and standard deviations (for both $\pm 10\%$ and $\pm 25\%$) of the percentage changes for each optimal controller. Percentages marked on the plots indicate the standard deviations across the entire Pareto front when parameters were varied by $\pm 10\%$ ($\pm 25\%$). Only original controllers where $\tau_{\pm 10} = \tau_{90}$ are considered.

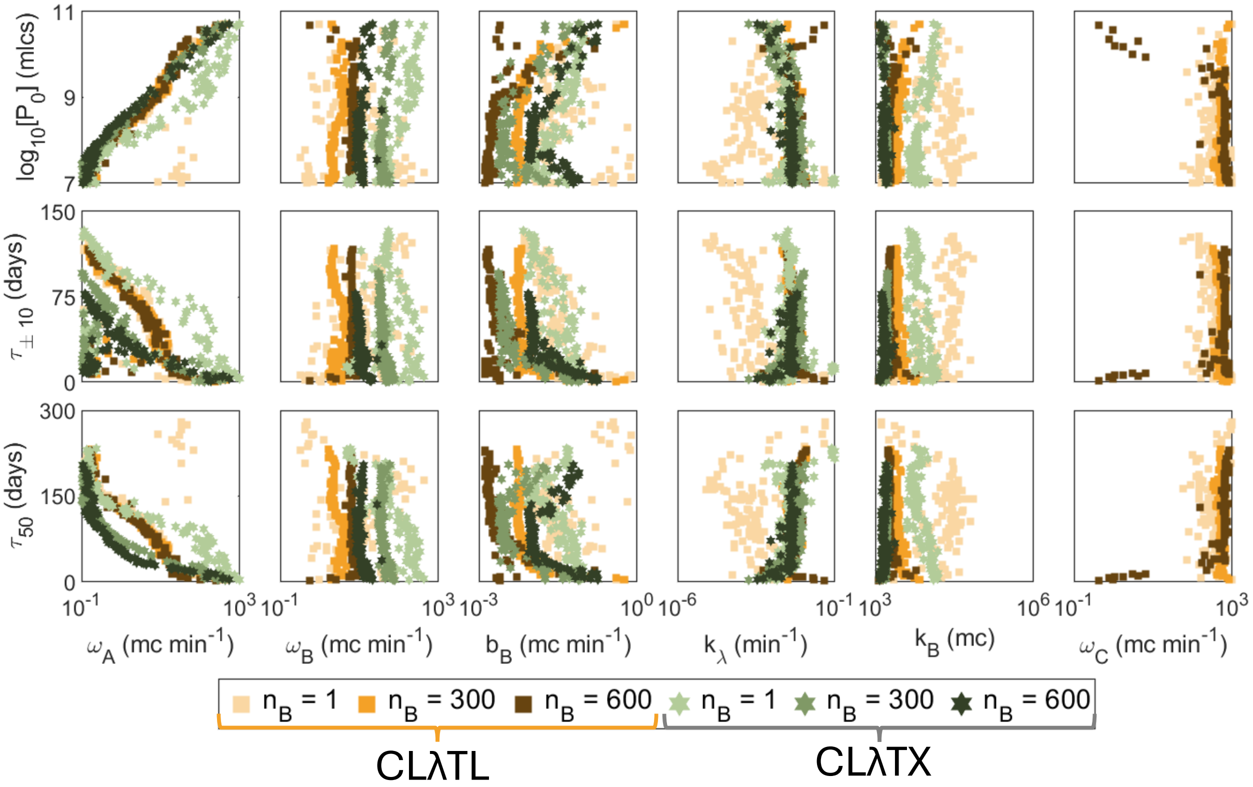

Supplementary Figure 17: Optimal parameter choices for growth-based control designs. We performed multi-objective optimisations on CLλTX (olive) and CLλTL (orange) for three controller sizes ($n_B = 1, 300, 600$ aa) to simultaneously maximise P_0, $\tau_{\pm 10}$ and τ_{50}. These three objectives are plotted against the optimal parameter choices for each system (ω_A, ω_B, b_B, k_λ, k_B and ω_C), corresponding to outputs in Fig. S16. (Note ω_C is not included in the model for CLλTX.)

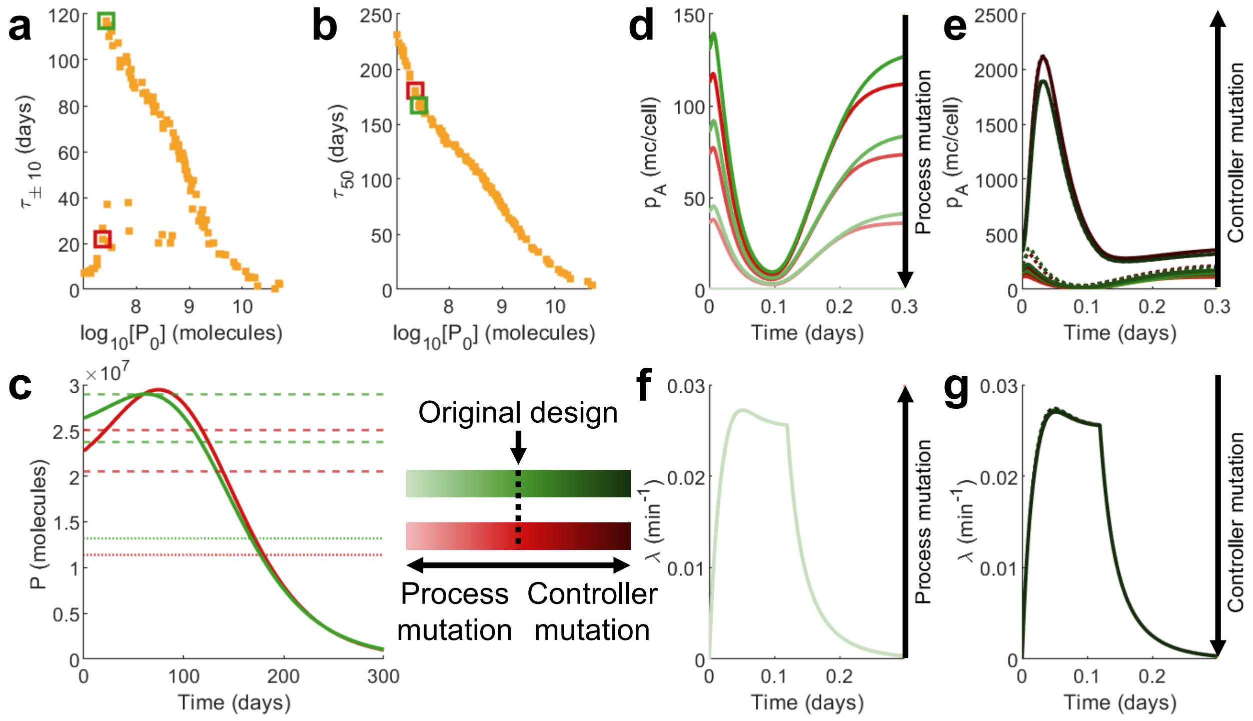

Supplementary Figure 18: Investigating why CLλTL can have poor $\tau_{\pm 10}$ performance at low initial outputs P_0. (a) $\tau_{\pm 10}$ and (b) τ_{50} against initial output P_0 for optimal controllers. Two specific designs are highlighted in green and red. (c) Time series outputs for the highlighted controller designs. Dashed lines show the window of $\pm 10\%$ of P_0 and dotted lines show $P_0/2$. (d) Protein output per cell p_A over time for the first day of simulation according to mutation state, considering only states with mutated processes. (e) Protein output per cell p_A over time for the first day of simulation, considering only states with mutated controllers (solid line mutation in ω_B, dotted line mutation in ω_C). (f) Growth rate λ over time for the first day of simulation according to mutation state, considering only states with mutated processes. (g) Growth rate λ over time for the first day of simulation, considering only states with mutated controllers (solid line mutation in ω_B, dotted line mutation in ω_C).

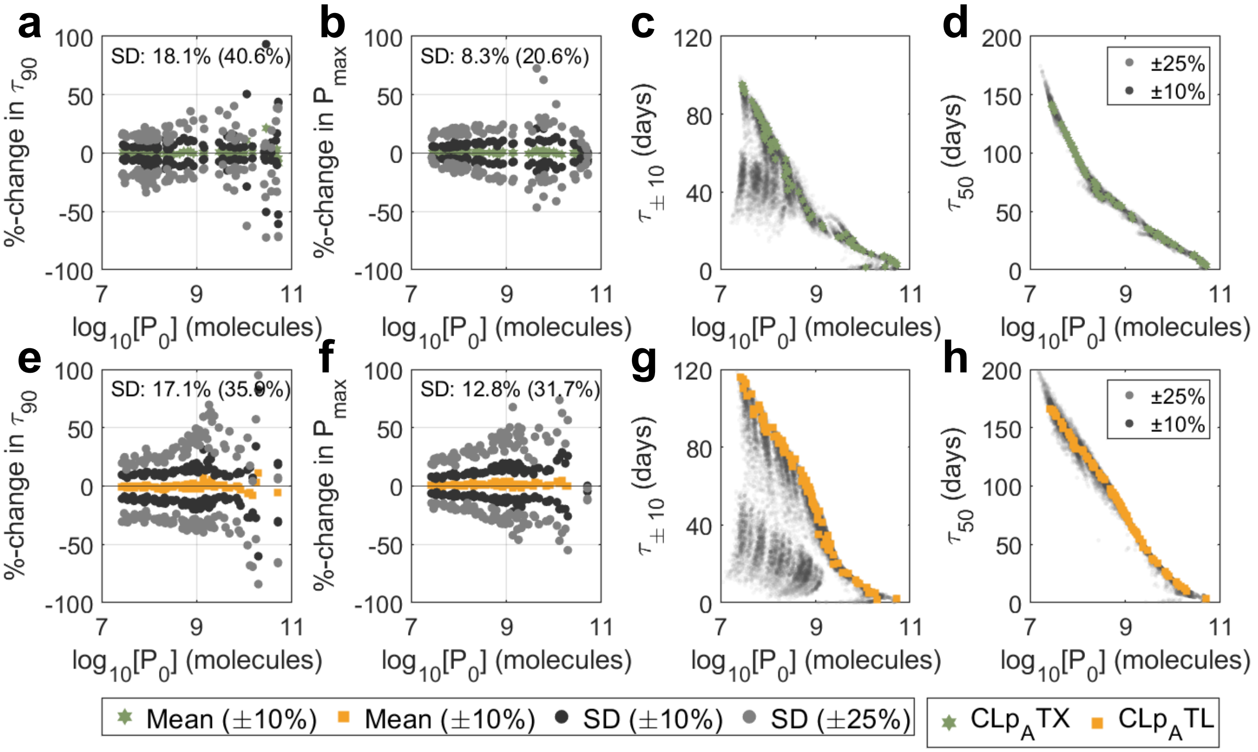

Supplementary Figure 19: Robustness analysis for (a-d) CLλTX (olive) and (e-h) CLλTX (orange). 100 optimal controllers for each system were generated by multi-objective optimisation with $n_B = 300$ aa. For each of these, 100 further controllers were generated by varying parameters by up to $\pm 10\%$ (dark grey) and $\pm 25\%$ (light grey). (a,b,e,f) The percentage changes in two output metrics were calculated versus the original optimal systems: (a,e) τ_{90} and (b,f) P_{max}. Plots show the means (for $\pm 10\%$) and standard deviations (for both $\pm 10\%$ and $\pm 25\%$) of the percentage changes for each optimal controller. Percentages marked on the plots indicate the standard deviations across the entire Pareto front when parameters were varied by $\pm 10\%$ ($\pm 25\%$). (c,d,g,h) The impact of parameter variation on the Pareto front. Coloured markers indicate the original front which simultaneously maximises P_0, $\tau_{\pm 10}$ and τ_{50}. Grey markers indicate systems with varied parameters. Plots show (c,g) $\tau_{\pm 10}$ and (d,h) τ_{50} against initial output P_0. Only original controllers where $\tau_{\pm 10} = \tau_{90}$ are considered.

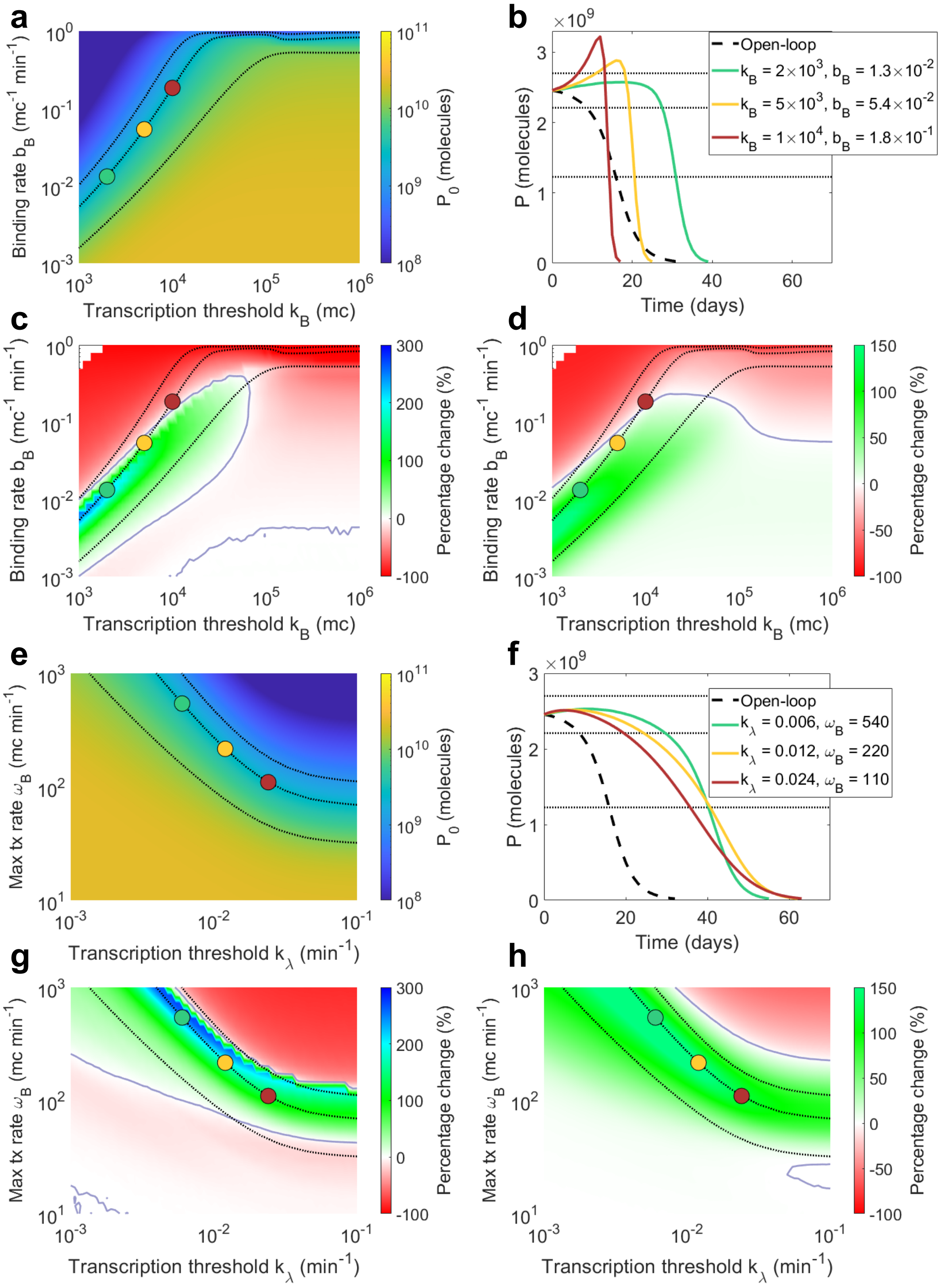

Supplementary Figure 20: Designing a transcriptional intra-circuit controller CLλTX for a nominal process with $\omega_A = 50$ mc min^{-1}. (a-d) $\omega_B = 100$ mc min^{-1}, $k_\lambda = 0.006$ min^{-1}. Parameters k_B and b_B vary. (e-h) $k_B = 10^3$ mc, $b_B = 0.001$ mc^{-1} min^{-1}. Parameters ω_B and k_λ vary. (a,e) Heatmap of initial protein output P_0 for different parameter combinations. Contours plotted for 1×10^9, 2.5×10^9, 1×10^{10} mc. Markers correspond to parameterizations in (b,f). (b,f) Time series protein output for three different controller parameterizations that each produce the same initial output. The dotted line marks the open-loop system. Red lines describe systems with weaker, more abundant controllers. Green lines describe systems with stronger, less abundant controllers. (c,d,g,h) Heatmap showing the percentage change in (c,g) $\tau_{\pm10}$ and (d,h) τ_{50} for different parameter combinations. Green and blue areas represent an improvement with feedback, red areas represent a worsening, and white areas represent little change. The blue contour marks the boundary where there is no change. Black contours are analogous to those in (a,e). Markers correspond to parameterizations in (b,f).

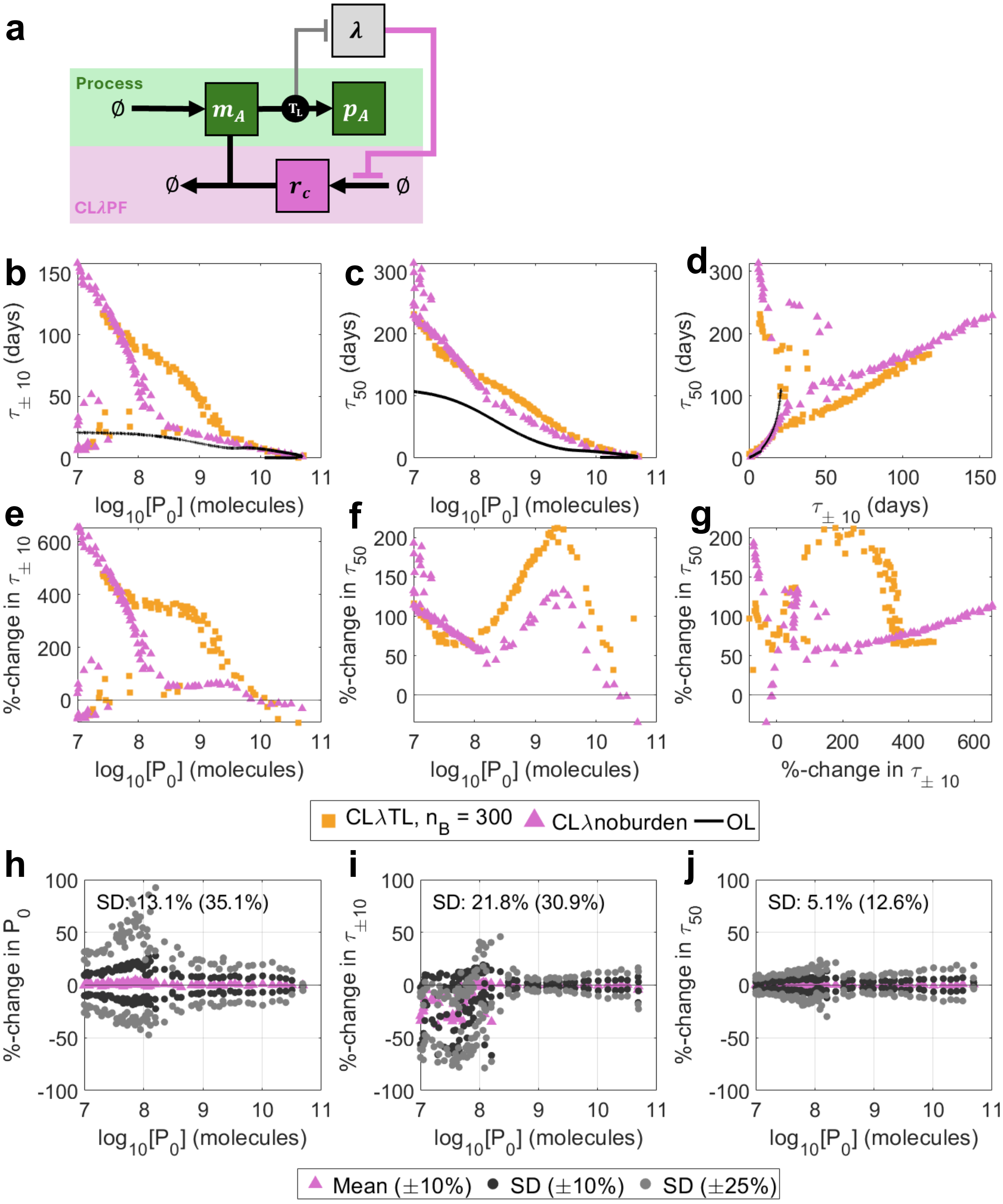

Supplementary Figure 21: Considering CLλprotein-free and comparing its performance against CLλTL. (a) A schematic describing CLλprotein-free. sRNA r_C is produced from a growth-sensitive promoter and silences process mRNA m_A. See key in Fig. 2a for symbol meanings. (b-g) Optimal performance for both CLλTL with $n_B = 300$ aa (orange) and CLλTX (pink). (b) $\tau_{\pm 10}$ vs initial output P_0, (c) τ_{50} vs initial output P_0, (d) τ_{50} vs $\tau_{\pm 10}$, (e) %-change in $\tau_{\pm 10}$ over open-loop vs initial output P_0, (f) %-change in τ_{50} over open-loop vs initial output P_0, (g) %-change in $\tau_{\pm 10}$ over open-loop vs %-change in τ_{50} over open-loop. (h-j) Robustness analysis. For each of the 100 optimal controllers for $n_B = 300$ aa, 100 further controllers were generated by varying parameters by up to ±10% (dark grey) and ±25% (light grey). The percentage changes in three output metrics were calculated versus the original optimal systems: (h) P_0, (i) $\tau_{\pm 10}$ and (j) τ_{50}. Plots show the means (for ±10%) and standard deviations (for both ±10% and ±25%) of the percentage changes for each optimal controller. Percentages marked on the plots indicate the standard deviations across the entire Pareto front when parameters were varied by ±10% (±25%). Only original controllers where $\tau_{\pm 10} = \tau_{90}$ are considered.

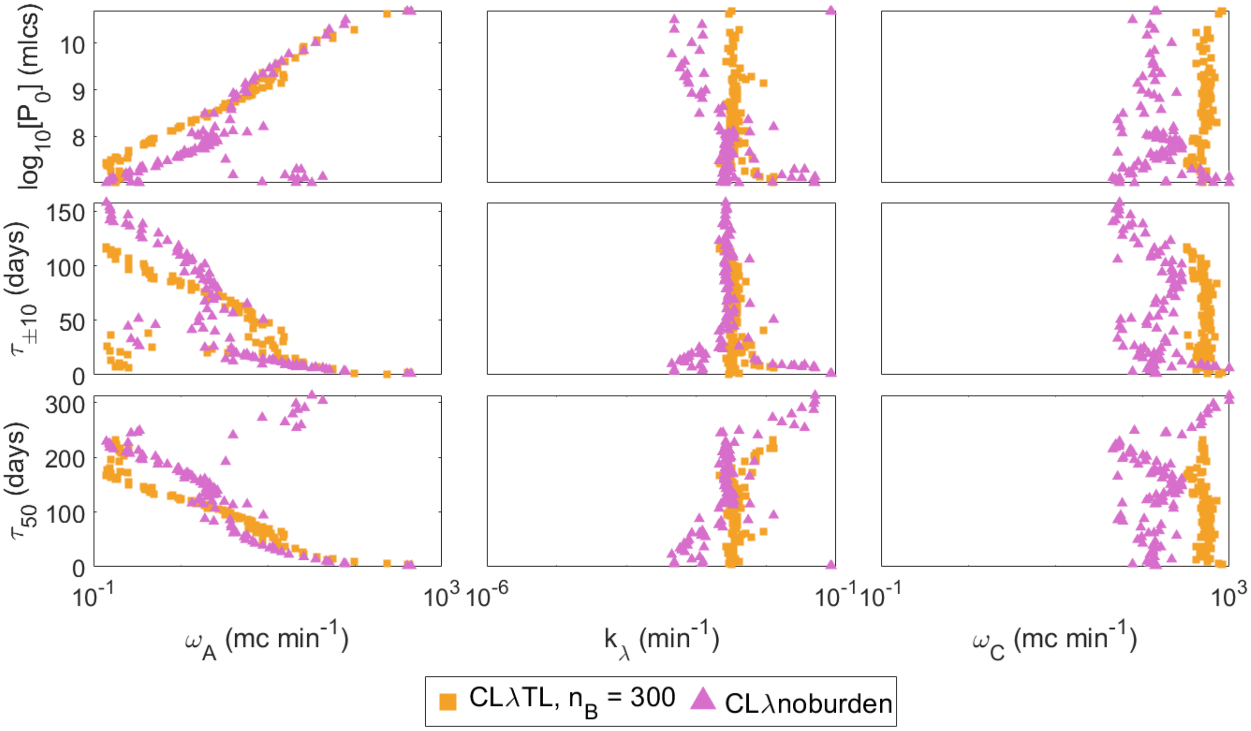

Supplementary Figure 22: Optimal parameter choices for protein-free growth-based control designs. We performed multi-objective optimisations on CLλprotein-free (pink) to simultaneously maximise P_0, $\tau_{\pm 10}$ and τ_{50}. These three objectives are plotted against the optimal parameter choices for each system (ω_A, ω_B, b_D, b_B and k_{BD}), corresponding to outputs in Fig. S21. Parameters are compared against those for CLλTL with $n_B = 300$ aa (orange).

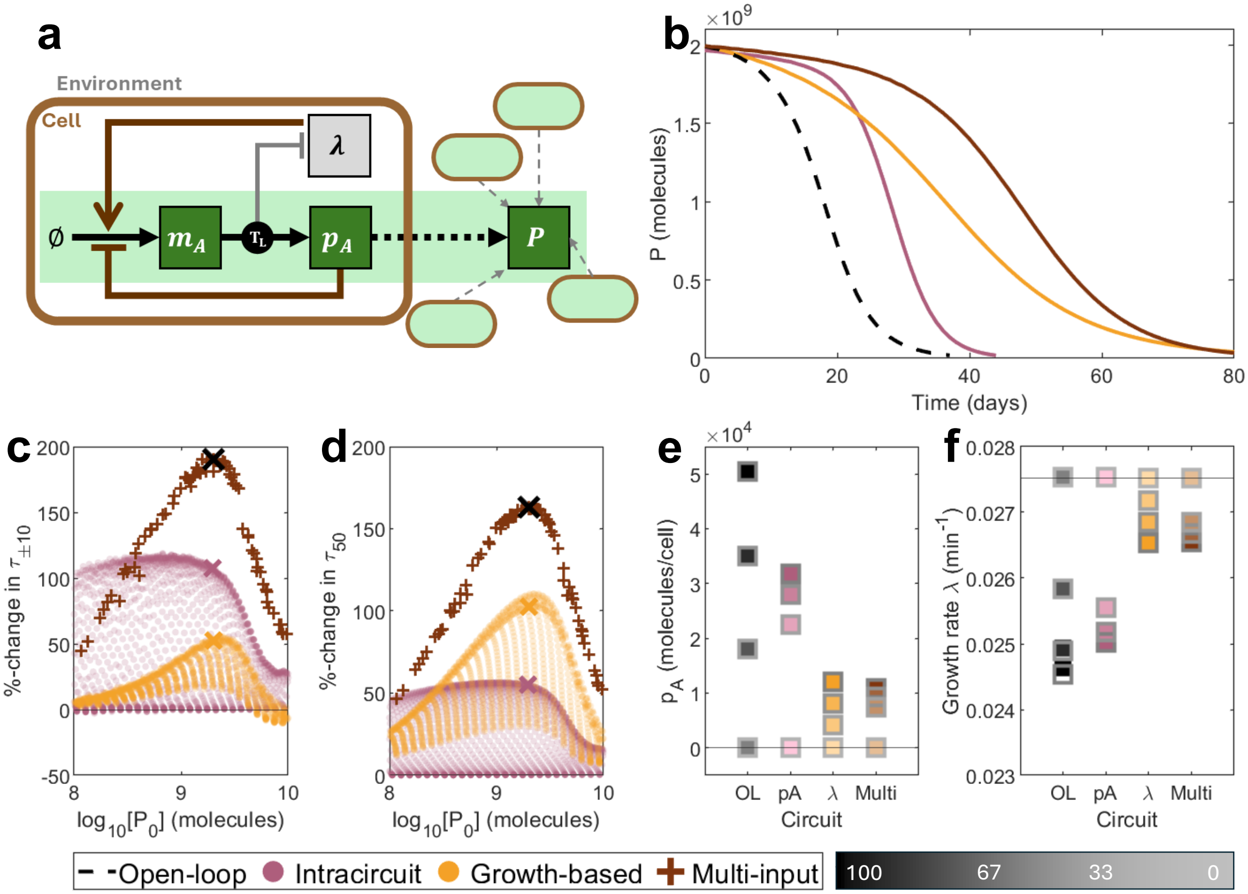

Supplementary Figure 23: Phenomenological modelling of multi-input control versus single-input control. (a) A schematic for a phenomenological model of a multi-input control system. The production of mRNA m_A is inhibited by synthetic protein production and activated by growth. (b) Time-series of population-wide output P over time for an open-loop system (black, dashed, $\omega_A = 4.0$ mc min^{-1}) and representative control systems of equivalent initial output P_0. (Intra-circuit: red, $w_A = 10^3$ mc min^{-1}, $k_A = 4.5 \times 10^2$ mc. Growth-based: orange, $w_A = 87$ mc min^{-1}, $k_\lambda = 6.3 \times 10^{-2}$ min^{-1}. Multi-input: brown, $w_A = 78$ mc min^{-1}, $k_A = 2.0 \times 10^3$ mc, $k_\lambda = 1.2 \times 10^{-3}$ min^{-1}.) (c)-(d) Percentage change in (c) $\tau_{\pm 10}$ and (d) τ_{50} versus an open-loop system of equivalent initial output P_0 for single- and multi-input controllers. Single-input controllers were generated by varying parameters. Multi-input controllers were generated using multi-objective optimisation as described in Methods 4.3. Points marked with an X correspond to the time-series plots in (b). These were selected as points on the Pareto fronts simultaneously optimising P_0, τ_{50} and $\tau_{\pm 10}$, with initial output P_0 closest to 2×10^9. (e-f) For circuits corresponding to trajectories in (b), (e) maximum protein production per cell p_A and (f) maximum growth rates λ across the first day (solid) and day where τ_{50} is reached (grey outline) for each mutation state. The horizontal line represents a non-functional strain.

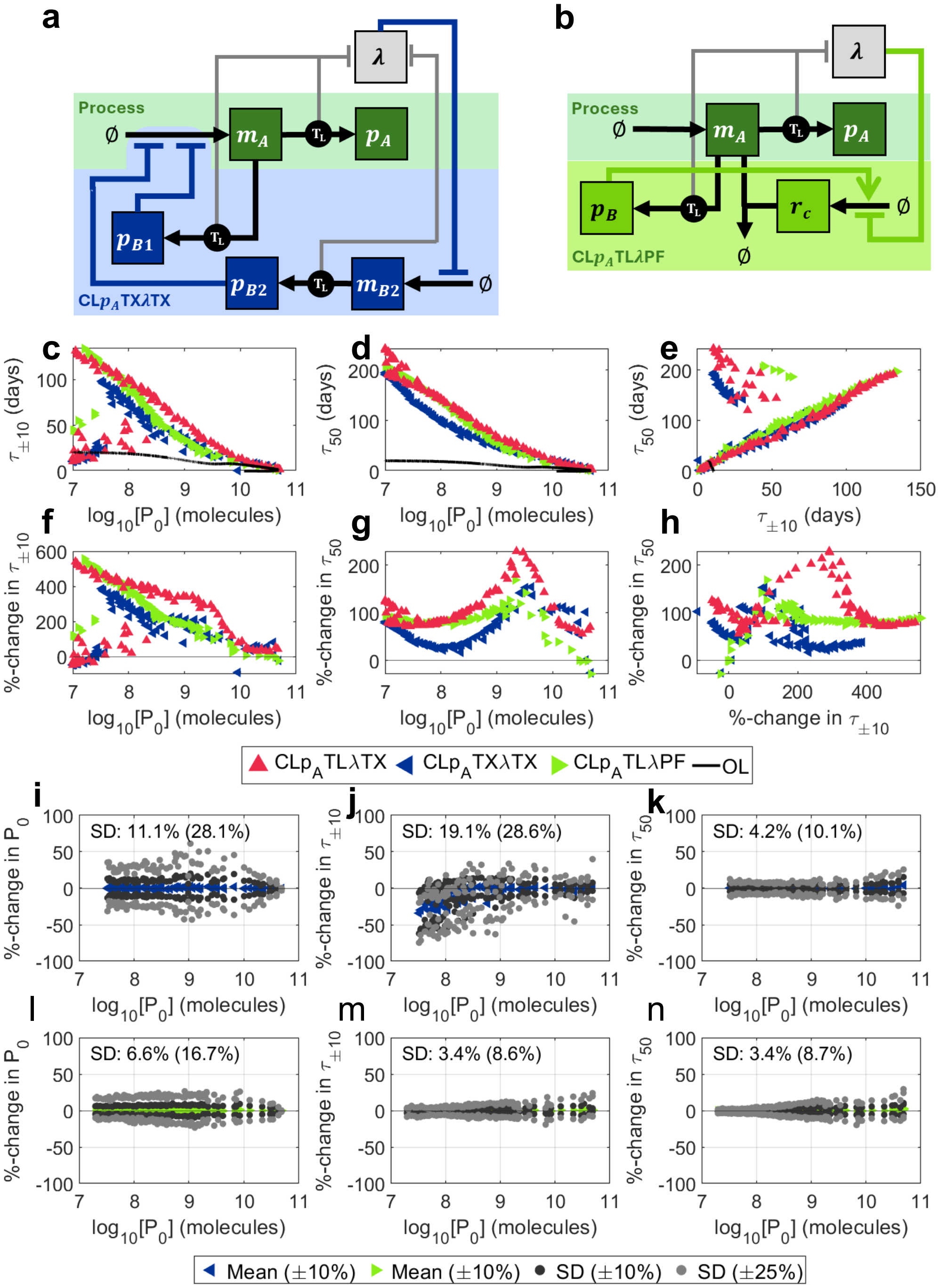

Supplementary Figure 24: Multi-input control design and performance. (a) A schematic describing the controller CLp_ATXλTX. Controller protein p_{B1} is produced from the same gene as output protein p_A. Controller protein p_{B2} is produced from a growth-sensitive promoter. Both p_{B1} and p_{B2} act as transcription factors to cooperatively inhibit the process. (b) A schematic describing the controller CLp_ATLλPF. A controller protein p_B is produced from the same gene as the output protein p_A, and acts as a transcription factor which activates the production of sRNA r_C, which is itself on a growth-sensitive promoter. The feedback loop is completed through the combining of r_C with mRNA that codes for p_A and p_B, preventing it from being translated. See key in Fig. 2a for symbol meanings. (c-h) Optimal performance for CLp_ATLλTX (crimson), CLp_ATXλTX (navy) and CLp_ATLλPF (lime) for controller protein length $n_B = 300$ aa. (c) $\tau_{\pm 10}$ vs initial output P_0, (d) τ_{50} vs initial output P_0, (e) τ_{50} vs $\tau_{\pm 10}$ (f) %-change in $\tau_{\pm 10}$ over open-loop vs initial output P_0, (g) %-change in τ_{50} over open-loop vs initial output P_0, (h) %-change in τ_{50} over open-loop vs %-change in $\tau_{\pm 10}$ over open-loop. (i-n) Robustness analyses for (i-k) CLp_ATXλTX and (l-n) CLp_ATLλPF. For each of the 100 optimal controllers, 100 further controllers were generated by varying parameters by up to ±10% (dark grey) and ±25% (light grey). The percentage changes in three output metrics were calculated versus the original optimal systems: (i,l) P_0, (j,m) $\tau_{\pm 10}$ and (k,n) τ_{50}. Plots show the means (for ±10%) and standard deviations (for both ±10% and ±25%) of the percentage changes for each optimal controller. Percentages marked on the plots indicate the standard deviations across the entire Pareto front when parameters were varied by ±10% (±25%).

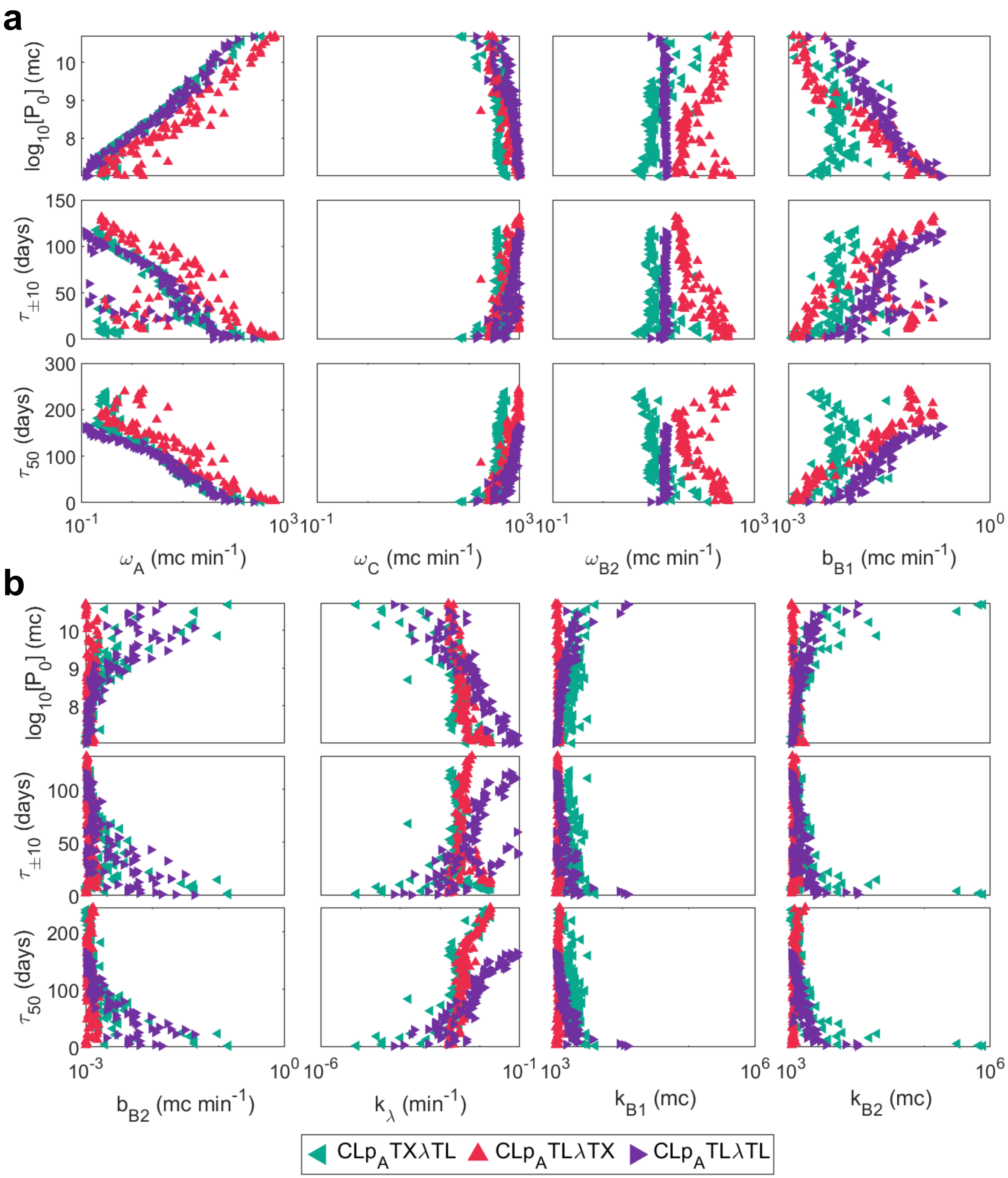

Supplementary Figure 25: Optimal parameter choices for multi-input controllers. We performed multi-objective optimisations on CLp_ATXλTL (teal), CLp_ATLλTX (crimson) and CLp_ATLλTL (purple) for n_B = 300 aa to simultaneously maximise $P0$, $\tau_{\pm 10}$ and τ_{50}. These three objectives are plotted against the optimal parameter choices for each system: (a) ω_A, ω_C, ω_D and b_B. (b) b_D, k_λ, k_B, k_D

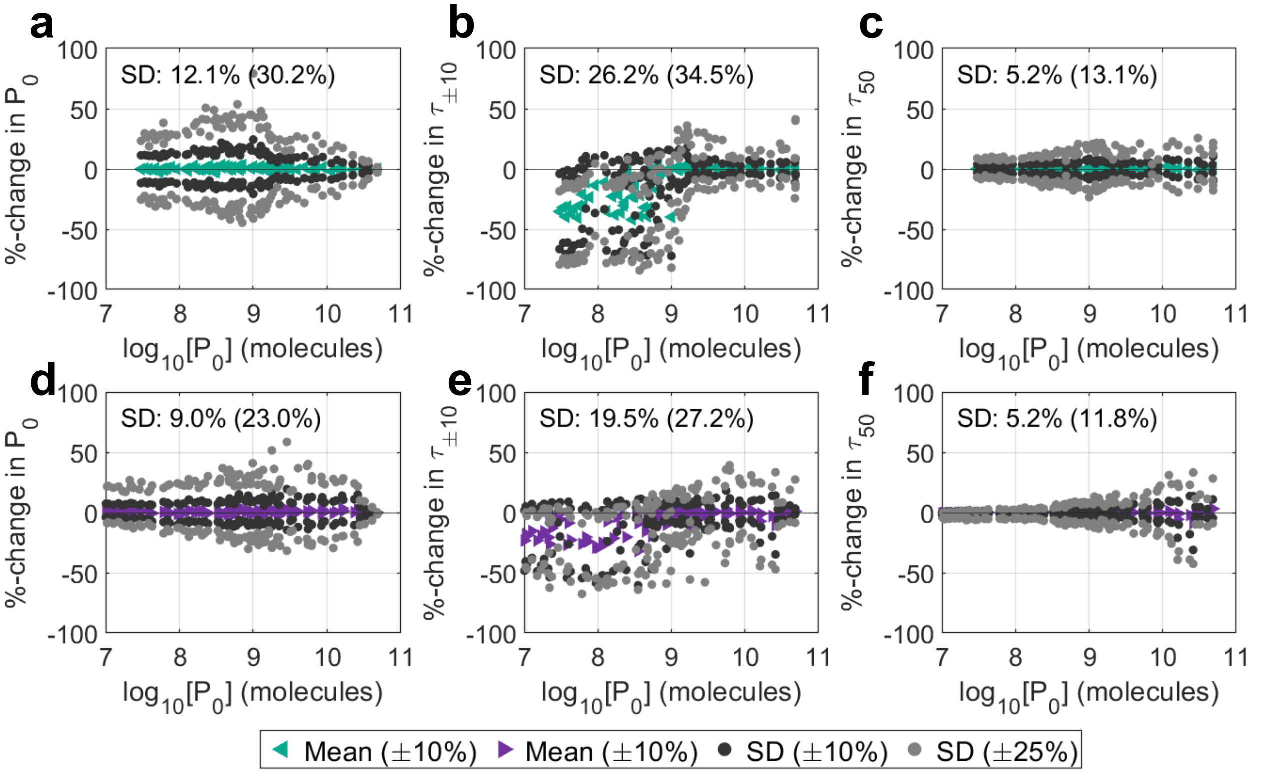

Supplementary Figure 26: Robustness analyses for (a-c) CLp_ATXλTL (teal) and (d-f) CLp_ATLλTL (purple). 100 optimal controllers for each system were generated by multi-objective optimisation with n_B = 300 aa. For each of these, 100 further controllers were generated by varying parameters by up to ±10% (dark grey) and ±25% (light grey). The percentage changes in three output metrics were calculated versus the original optimal systems: (a,d) τ_{P_0}, (b,e) $\tau_{\pm 10}$ and (c,f) τ_{50}. Plots show the means (for ±10%) and standard deviations (for both ±10% and ±25%) of the percentage changes for each optimal controller. Percentages marked on the plots indicate the standard deviations across the entire Pareto front when parameters were varied by ±10% (±25%).

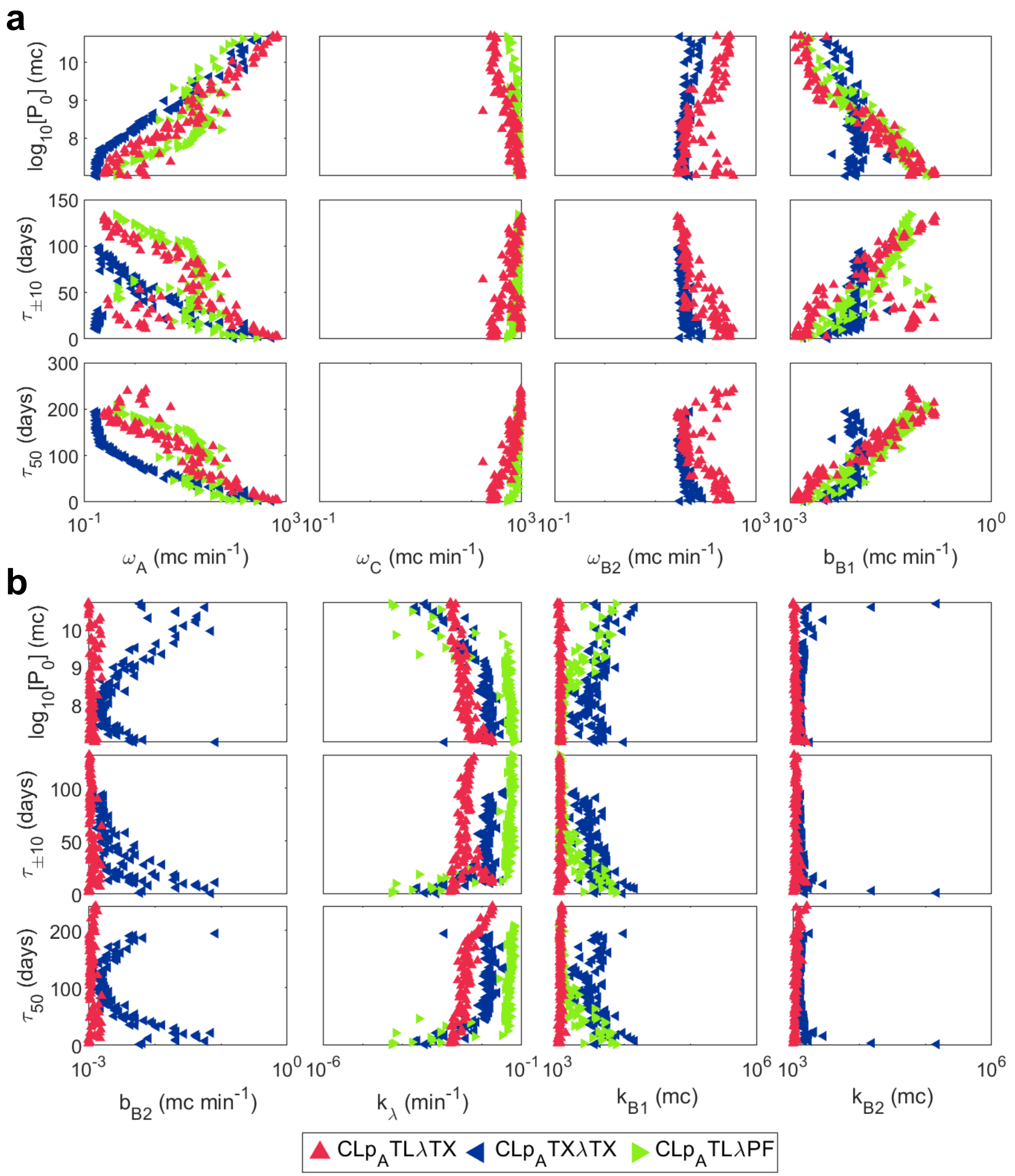

Supplementary Figure 27: Optimal parameter choices for multi-input controllers. We performed multi-objective optimisations on CLp_ATLλTX (crimson), CLp_ATXλTX (navy) and CLp_ATLλPF (lime) for n_B = 300 aa to simultaneously maximise $P0$, $\tau_{\pm 10}$ and τ_{50}. These three objectives are plotted against the optimal parameter choices for each system: (a) ω_A, ω_C, ω_D and b_B. (b) b_D, k_λ, k_B, k_D. Note that CLp_ATXλTX does not have the parameter ω_C and CLp_ATLλPF does not have the parameters ω_{B2} or k_{B2}.

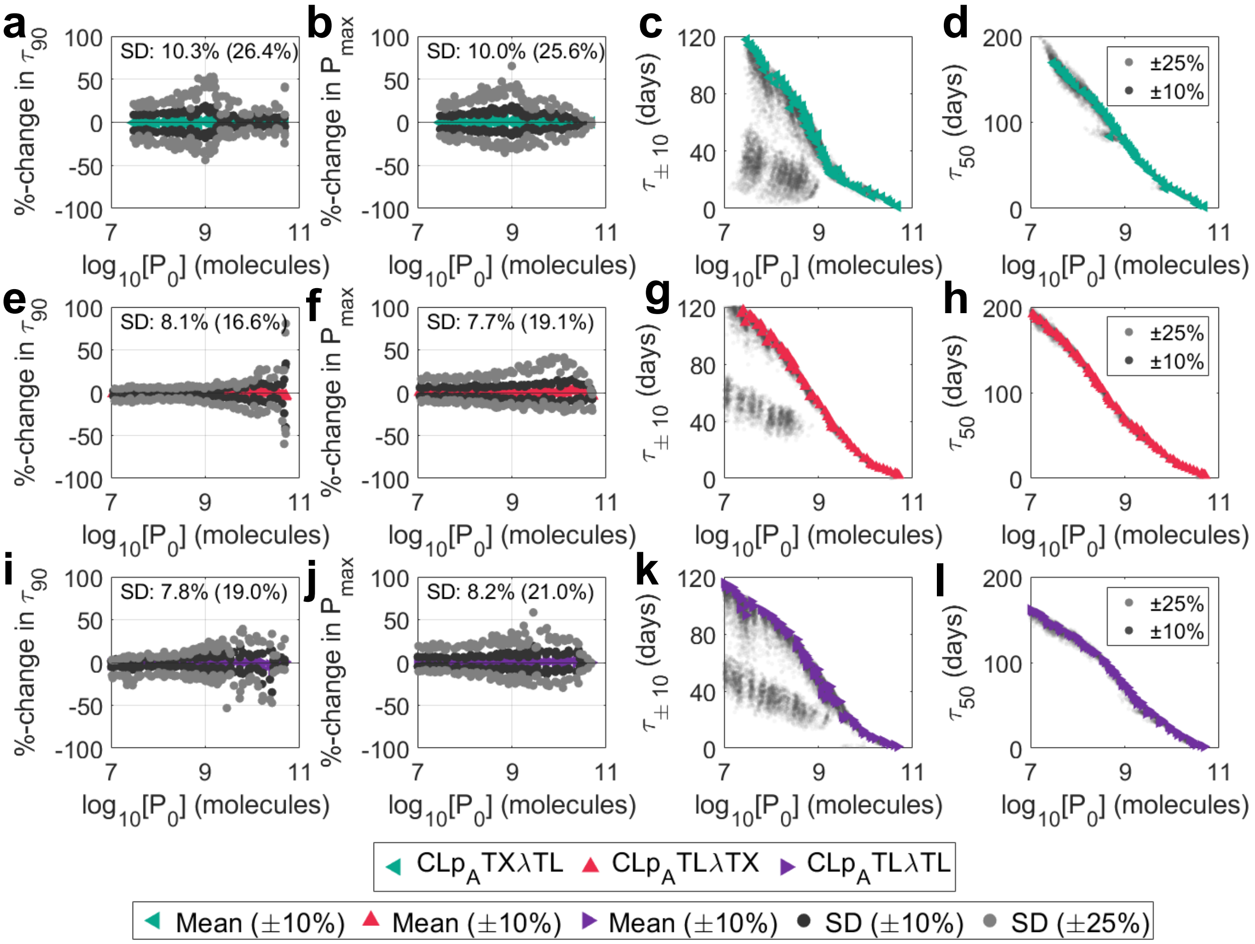

Supplementary Figure 28: Robustness analysis for (a-d) CLp_ATXλTL (teal), (e-h) CLp_ATLλTX (crimson), (i-l) CLp_ATLλTL (purple). 100 optimal controllers for each system were generated by multi-objective optimisation with $n_B = 300$ aa. For each of these, 100 further controllers were generated by varying parameters by up to ±10% (dark grey) and ±25% (light grey). (a,b,e,f,i,j) The percentage changes in two output metrics were calculated versus the original optimal systems: (a,e,i) τ_{90} and (b,f,j) P_{max}. Plots show the means (for ±10%) and standard deviations (for both ±10% and ±25%) of the percentage changes for each optimal controller. Percentages marked on the plots indicate the standard deviations across the entire Pareto front when parameters were varied by ±10% (±25%). (c,d,g,h,k,l) The impact of parameter variation on the Pareto front. Coloured markers indicate the original front which simultaneously maximises P_0, $\tau_{\pm 10}$ and τ_{50}. Grey markers indicate systems with varied parameters. Plots show (c,g,k) $\tau_{\pm 10}$ and (d,h,l) τ_{50} against initial output P_0. Only original controllers where $\tau_{\pm 10} = \tau_{90}$ are considered.

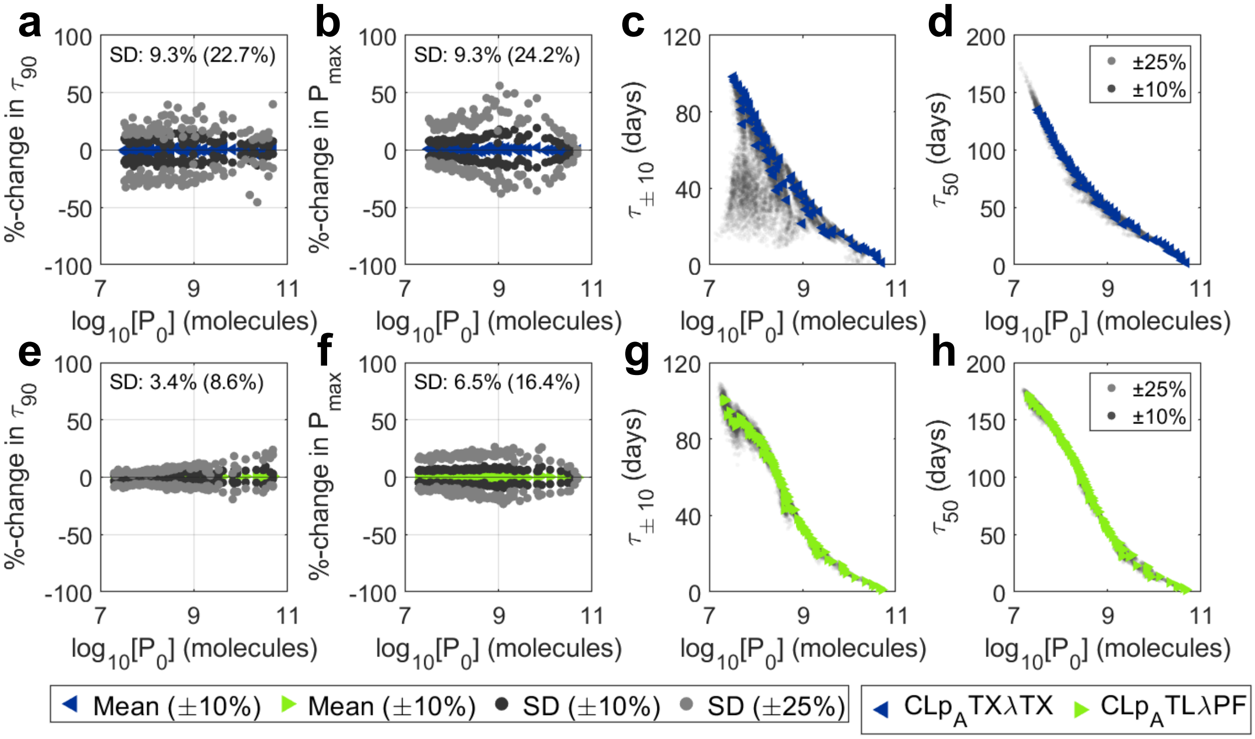

Supplementary Figure 29: Robustness analysis for (a-d) CLp_ATXλTX (navy) and (e-h) CLp_ATXλPF (lime). 100 optimal controllers for each system were generated by multi-objective optimisation with $n_B = 300$ aa. For each of these, 100 further controllers were generated by varying parameters by up to ±10% (dark grey) and ±25% (light grey). (a,b,e,f) The percentage changes in two output metrics were calculated versus the original optimal systems: (a,e) τ_{90} and (b,f) P_{max}. Plots show the means (for ±10%) and standard deviations (for both ±10% and ±25%) of the percentage changes for each optimal controller. Percentages marked on the plots indicate the standard deviations across the entire Pareto front when parameters were varied by ±10% (±25%). (c,d,g,h) The impact of parameter variation on the Pareto front. Coloured markers indicate the original front which simultaneously maximises P_0, $\tau_{\pm10}$ and τ_{50}. Grey markers indicate systems with varied parameters. Plots show (c,g) $\tau_{\pm10}$ and (d,h) τ_{50} against initial output P_0. Only original controllers where $\tau_{\pm10} = \tau_{90}$ are considered.

Supplementary Tables

Name	Control input	Control mechanism	d
CLp_ATX	Controller protein produced on the same gene as output protein	Inhibitory transcription factor	1
CLp_ATL	Controller protein produced on the same gene as output protein	sRNA	2
CLλTX	Cell growth rate λ	Inhibitory transcription factor	2
CLλTL	Cell growth rate λ	sRNA	3
CLλp-free	Cell growth rate λ	sRNA	1
CLQSTX	Quorum sensing molecule	Inhibitory transcription factor	2
CLp_ATXλTL	Controller protein produced on the same gene as output protein	Inhibitory transcription factor	3
	Cell growth rate λ	sRNA	
CLp_ATLλTX	Controller protein produced on the same gene as output protein	sRNA	3
	Cell growth rate λ	Inhibitory transcription factor	
CLp_ATLλTL	Controller protein produced on the same gene as output protein	sRNA	3
	Cell growth rate λ	sRNA	
CLp_ATXλTX	Controller protein produced on the same gene as output protein	Inhibitory transcription factor	2
	Cell growth rate λ	Inhibitory transcription factor	
CLp_ATLλPF	Controller protein produced on the same gene as output protein	sRNA	2
	Cell growth rate λ	sRNA	

Supplementary Table 1: An outline of the different controller topologies used throughout the paper.

±10%	Mean (%-change vs original)					σ (%-change vs original)					Points with
	P_0	τ_{50}	$\tau_{\pm 10}$	τ_{90}	P_{max}	P_0	τ_{50}	$\tau_{\pm 10}$	τ_{90}	P_{max}	$\tau_{\pm 10} = \tau_{90}$ (%)
CLp_ATX	0.21	0.04	-0.22	-0.22	0.21	5.56	2.86	3.26	3.26	5.56	100
CLp_ATX†	0.31	0.05	-0.06	-0.06	0.31	5.54	3.80	3.76	3.76	5.54	100
CLp_ATL	0.63	-0.03	-0.14	-0.14	0.58	7.32	4.27	4.55	4.55	7.19	100
CLλTX	0.04	-0.07	-4.58	0.79	0.32	9.10	9.18	22.65	18.09	8.32	88.86
CLλTL	1.04	-0.64	-18.10	-0.19	1.08	15.30	9.17	30.78	17.05	12.75	78.47
CLλPF	0.86	0.06	-9.15	-0.02	0.76	13.07	5.14	21.84	9.97	11.22	87.57
CLQSTX	0.71	-0.00	-9.42	-0.43	0.83	12.31	5.85	21.55	10.66	10.28	86.53
CLp_ATXλTL	0.30	0.09	-12.75	0.23	0.26	12.11	5.19	26.18	10.31	9.98	83.69
CLp_ATLλTX	0.44	-0.25	-5.18	-0.26	0.43	8.05	6.77	16.92	8.14	7.65	91.17
CLp_ATLλTL	0.59	-0.32	-7.79	-0.54	0.55	8.97	5.19	19.54	7.82	8.15	88.25
CLp_ATXλTX	0.18	0.12	-7.55	-0.26	0.51	11.05	4.18	19.06	9.31	9.27	87.62
CLp_ATLλPF	0.27	0.00	-0.01	-0.01	0.26	6.63	3.41	3.37	3.37	6.51	100

±25%	Mean (%-change vs original)					σ (%-change vs original)					Points with
	P_0	τ_{50}	$\tau_{\pm 10}$	τ_{90}	P_{max}	P_0	τ_{50}	$\tau_{\pm 10}$	τ_{90}	P_{max}	$\tau_{\pm 10} = \tau_{90}$ (%)
CLp_ATX	0.85	0.30	-0.13	-0.13	0.85	13.92	6.95	6.84	6.84	13.92	100
CLp_ATX†	1.25	0.29	-0.41	-0.41	1.25	13.98	8.17	9.64	9.64	13.98	100
CLp_ATL	3.90	-0.41	-0.73	-0.73	3.61	18.66	10.73	11.62	11.61	18.35	99.99
CLλTX	0.35	0.53	-9.30	0.94	1.70	22.57	17.35	43.90	40.59	20.64	83.24
CLλTL	3.50	-0.38	-31.40	-0.40	4.24	37.30	23.97	38.66	35.90	31.65	71.80
CLλPF	5.63	0.38	-18.42	0.38	5.38	35.08	12.58	30.85	24.04	31.01	80.05
CLQSTX	2.91	0.58	-20.51	-0.20	4.49	31.40	14.33	33.21	25.78	26.80	76.32
CLp_ATXλTL	1.82	0.71	-22.05	2.17	2.36	30.24	13.12	34.54	26.41	25.61	76.73
CLp_ATLλTX	3.97	-1.10	-9.60	-1.27	3.82	20.00	14.39	24.56	16.58	19.09	86.64
CLp_ATLλTL	3.71	-1.07	-13.69	-2.40	3.28	22.95	11.77	27.22	19.02	21.03	84.30
CLp_ATXλTX	2.09	-0.06	-17.00	-1.96	3.54	28.12	10.14	28.59	22.67	24.23	80.61
CLp_ATLλPF	2.20	-0.21	-0.42	-0.42	2.14	16.69	8.65	8.59	8.59	16.43	100

Supplementary Table 2: Comparing the robustness of different controllers. For the 100 optimal controller designs on each Pareto front, we first discarded those with poor short-term performance due to $\tau_{\pm 10} \neq \tau_{90}$. For each of the remaining designs, we generated 100 new designs where each designable parameter was varied by up to ±10% (upper) and ±25% (lower) of its original value, and calculated the percentage change in various parameters vs the original design. The table presents the mean and standard deviations of these percentage changes, as well as the percentage of designs for which $\tau_{\pm 10} = \tau_{90}$ is maintained. Key robustness metrics for CLλTL are highlighted in red, and the best performing ones for the multi-input controllers considered in the main text are highlighted in blue. † CLp_ATX with mutation in the k_B parameter.

Variable	Description	Units	No.	
s_X	External substrate	mc	1	Mutation model
N	Population size	-	n	Population model
s_I	Internal substrate	mc/cell	n	Host model
e	Energy	mc/cell	n	Host model
$m_{\{T,E,H,R\}}$	Host gene mRNA	mc/cell	n	Host model
$c_{\{T,E,H,R\}}$	Host gene mRNA-ribosome translating complex	mc/cell	n	Host model
$p_{\{T,E,H,R\}}$	Host gene protein	mc/cell	n	Host model
r	rRNA	mc/cell	n	Host model
R	Functional ribosome	mc/cell	n	Host model
m_A	Synthetic gene mRNA	mc/cell	n	Circuit model
c_A	Synthetic gene mRNA-ribosome translating complex	mc/cell	n	Circuit model
p_A	Synthetic gene protein	mc/cell	n	Circuit model

Supplementary Table 3: Variables for the combined multi-scale model. For the open-loop system, there are $20n + 1$ total variables, where n is the number of mutation states. Here, mc := molecules.

Parameter	Description	Value	Units	Ref
ω_A	Maximal circuit transcription rate	$[10^{-1}, 10^3]$	mc min^{-1}	$*$
π_A	Circuit gene transcription energy threshold	4.38	mc	†
n_A	Circuit protein length	300	aa	†
b_A	Circuit mRNA-ribosome binding rate	0.1	mc min^{-1}	∇
u_A	Circuit mRNA-ribosome unbinding rate	0.01	min-1	∇
δ_{m_A}	Circuit mRNA degradation rate	0.1	min^{-1}	†

Supplementary Table 4: Parameters for the synthetic process. * Parameter varies over a large biologically feasible range. † Assumed to be equal to host parameters. ∇ Biologically sensible choices, similar to those used in [1].

Parameter	Description	Value	Units	Ref
ϕ_e	Nutrient efficiency	20	-	†
v_T	Maximal nutrient import	726	mc min^{-1}	[2]
v_E	Maximal substrate-to-energy conversion	5800	mc min^{-1}	[2]
$k_{\{T,E\}}$	Transporter/enzyme Michaelis-Menten constant	10^3	mc	[2]
$\omega_{\{T,E\}}$	Maximal transport/enzyme transcription rate	4.14	mc min^{-1}	[2]
ω_H	Maximal general host protein transcription rate	949	mc min^{-1}	[2]
ω_R	Maximal ribosomal mRNA transcription rate	930	mc min^{-1}	[2]
ω_r	Maximal rRNA transcription rate	3170	mc min^{-1}	[2]
$o_{\{T,E,H\}}$	Host genes transcription energy threshold	4.38	mc	[2]
$o_{\{R,r\}}$	Ribosomal genes transcription energy threshold	427	mc	[2]
$n_{\{T,E,H\}}$	Host genes protein length	300	aa	[2]
n_R	Ribosomal protein length	7459	aa	[2]
$b_{\{T,E,H,R\}}$	mRNA-ribosome binding rate	0.95×10^{-2}	mc min^{-1}	[1]
$u_{\{T,E,H,R\}}$	mRNA-ribosome unbinding rate	1	min^{-1}	[2]
$\delta_{m_{\{T,E,H,R\}}}$	Host mRNA degradation rate	0.1	min^{-1}	[2]
δ_r	rRNA degradation rate	0.1	min^{-1}	[2]
b_ρ	Ribosome formation rate (from protein and rRNA)	1	mc min^{-1}	[2]
u_ρ	Ribosome unbinding rate (into protein and rRNA)	1	min^{-1}	[2]
$\gamma_{\max}$	Maximal elongation rate	1260	aa min^{-1}	[2]
k_γ	Elongation energy threshold	8×10^8	mc	†
M_0	Proteome size	10^8	aa	[2]
k_H	Host protein transcription threshold	152219	mc	[2]
h_H	Host protein transcription Hill constant	4	-	[2]

Supplementary Table 5: Parameters for the *E. coli* host model. Most parameters are taken directly from previous models. Those marked with a † were selected to ensure that a representative system reaches steady state within $\approx$ 8 to 12 hours, corresponding to realistic repeated batch experiments [3]. These parameters were originally defined in [4] and were similarly modified when modelling populations.

ω_A (%)	100	67	33	0
100	-	σ	σ^2	σ^3
67	-	-	σ	σ^2
33	-	-	-	σ
0	-	-	-	-

Supplementary Table 6: A matrix showing the mutation scheme for dimension $d = 1$ and states per dimension $s = 4$.

ω_A<br>ω_B (%)	100<br>100	100<br>67	100<br>33	100<br>0	67<br>100	67<br>67	67<br>33	67<br>0	33<br>100	33<br>67	33<br>33	33<br>0	0<br>100	0<br>67	0<br>33	0<br>0
100<br>100	-	σ	σ^2	σ^3	σ	-	-	-	σ^2	-	-	-	σ^3	-	-	-
100<br>67	-	-	σ	σ^2	-	σ	-	-	-	σ^2	-	-	-	σ^3	-	-
100<br>33	-	-	-	σ	-	-	σ	-	-	-	σ^2	-	-	-	σ^3	-
100<br>0	-	-	-	-	-	-	-	σ	-	-	-	σ^2	-	-	-	σ^3
67<br>100	-	-	-	-	-	σ	σ^2	σ^3	σ	-	-	-	σ^2	-	-	-
67<br>67	-	-	-	-	-	-	σ	σ^2	-	σ	-	-	-	σ^2	-	-
67<br>33	-	-	-	-	-	-	-	σ	-	-	σ	-	-	-	σ^2	-
67<br>0	-	-	-	-	-	-	-	-	-	-	-	σ	-	-	-	σ^2
33<br>100	-	-	-	-	-	-	-	-	-	σ	σ^2	σ^3	σ	-	-	-
33<br>67	-	-	-	-	-	-	-	-	-	-	σ	σ^2	-	σ	-	-
33<br>33	-	-	-	-	-	-	-	-	-	-	-	σ	-	-	σ	-
33<br>0	-	-	-	-	-	-	-	-	-	-	-	-	-	-	-	σ
0<br>100	-	-	-	-	-	-	-	-	-	-	-	-	-	σ	σ^2	σ^3
0<br>67	-	-	-	-	-	-	-	-	-	-	-	-	-	-	σ	σ^2
0<br>33	-	-	-	-	-	-	-	-	-	-	-	-	-	-	-	σ
0<br>0	-	-	-	-	-	-	-	-	-	-	-	-	-	-	-	-

Supplementary Table 7: A matrix showing the mutation scheme for dimension $d = 2$ and states per dimension $s = 4$.

Par.	Description	Value	Units	Ref	Used
ω_A	Process maximal transcription rate	$[10^{-1}, 10^3]$	mc min^{-1}	$*$	1-6
o_A	Process transcription energy threshold	4.38	mc	†	1-6
n_A	Process protein length	300	aa	†	1-6
b_A	Process mRNA-ribosome binding rate	0.1	mc min^{-1}	∇	1-6
u_A	Process mRNA-ribosome unbinding rate	0.01	min^{-1}	∇	1-6
δ_{mA}	Process mRNA degradation rate	0.1	min^{-1}	†	1-6
ω_B	Transcription factor maximal transcription rate	$[10^{-1}, 10^3]$	mc min^{-1}	$*$	3-4
o_B	Transcription factor transcription energy threshold	4.38	mc	†	3-4
n_B	Transcription factor protein length	1,300,600	aa	‡	1-4,6
b_B	Transcription factor mRNA-ribosome binding rate	$[10^{-3}, 10^0]$	mc min^{-1}	$*$	1-4,6
u_B	Transcription factor mRNA-ribosome unbinding rate	0.01	min^{-1}	∇	1-4,6
k_B	Transcription factor control threshold	$[10^3, 10^6]$	mc	$*$	1-4
ω_C	sRNA maximal transcription rate	$[10^{-1}, 10^3]$	mc min^{-1}	$*$	2,4,5
o_C	sRNA transcription energy threshold	4.38	mc	†	2,4,5
δ_{rC}	sRNA degradation rate	0.1	min^{-1}	†	2,4,5
a_C	Process mRNA-sRNA annihilation rate	0.1	mc min^{-1}	§	2,4,5
k_λ	Growth-sensitive promoter threshold	$[10^{-6}, 10^{-1}]$	min^{-1}	$*$	3-5
ω_D	Synthetic enzyme maximal transcription rate	$[10^{-1}, 10^3]$	mc min^{-1}	$*$	6
o_D	Synthetic enzyme transcription energy threshold	4.38	mc	†	6
n_D	Synthetic enzyme length	1,300,600	aa	‡	6
b_D	Synthetic enzyme mRNA-ribosome binding rate	$[10^{-3}, 10^0]$	mc min^{-1}	$*$	6
u_D	Synthetic enzyme mRNA-ribosome unbinding rate	0.01	min^{-1}	∇	6
k_D	Synthetic enzyme Michaelis-Menten constant	10^3	mc	†	6
$v_{\max D}$	Synthetic enzyme maximal reaction rate	580	mc min^{-1}	‖	6
$v_{\exp D}$	Synthetic metabolite export rate	0.1	mc min^{-1}	§	6
ϕ_D	Synthetic enzyme energy consumption	0	-	‖	6
b_{BD}	Transcription factor-metabolite binding rate	0.1	mc min^{-1}	§	6
u_{BD}	Transcription factor-metabolite unbinding rate	0.01	min^{-1}	§	6
k_{BD}	Activated transcription factor control threshold	$[10^3, 10^6]$	mc	$*$	6
V_{cell}	Cell volume	10^{-7}	m^3	‖	6
V_{cult}	Culture volume	1	m^3	‖	6

Supplementary Table 8: Parameters used in the control systems. 1) CLp_ATX, 2) CLp_ATL, 3) CLλTX, 4) CLλTL, 5) CLλprotein-free, 6) CLQSTX. $*$ Varied over a wide biologically feasible range. † Assumed equal to host parameters. ∇ Biologically sensible choices, similar to those used in [1]. ‡ Separate analyses run for three different controller protein sizes. § Initial simulations sampled using EFAST [5] suggested these parameters have minimal influence on optimal performance, so fixed at biologically feasible values. ‖ Other: $v_{\max D}$ chosen so that synthetic enzymes are one tenth as efficient as native enzymes, ϕ_D set to 0 ignores additional burden from the consumption of small molecules to produce metabolites, V_{cell} and V_{cult} represent biologically feasible choices. In reality, V_{cell} is much smaller, but wide variation in magnitude negatively impacts simulation accuracy.

SUPPLEMENTARY NOTES

SN1 Supplementary Note 1: Full model description

Here we present a detailed description of the model used throughout this work. The complete model consists of four layers, where each layer is embedded in the next to produce a combined multi-scale model:

1. A circuit model, describing the dynamics of a synthetic circuit.
2. The host model, describing the dynamics of an *E. coli* cell.
3. The population model, describing the population dynamics of a set of host cells.
4. The mutation model, describing the dynamics of multiple competing populations which share a supply of substrate and can 'mutate' into each other.

The circuit, host and population models are built upon previous host-aware coarse-grained models of *E. coli* [4, 2]. The mutation model is similar to other recent models [6, 7]. Table S3 lists all the variables used in the combined model.

SN1.1 The open-loop synthetic circuit model

Throughout this paper, we consider a simple synthetic circuit that consists of a single gene A which produces output protein p_A. This process is modelled via three ordinary differential equations (ODEs) representing the following variables: mRNA m_A, translating mRNA-ribosome complexes c_A and proteins p_A. mRNA molecules m_A are spawned according to the transcription rate $T_{X_A}(e)$, dependent on energy e. The mRNA binds with host ribosomes R at a rate b_A to form translating complexes c_A and decays at a rate δ_{m_A}. The translating complexes produce proteins and release bound mRNA at the translation rate $T_{L_A}(c_A, e)$, dependent on the energy supply e and number of translating complexes c_A. Complexes can also unbind without completing translation at a rate u_A. The circuit model exploits the host energy e and ribosomes R. All variables are diluted by the host growth rate λ. This yields the following model:

$$\dot{m}_A = T_{X_A}(e) + T_{L_A}(c_A, e) - b_A R m_A + u_A c_A - (\lambda + \delta_{m_A}) m_A, \tag{1}$$

$$\dot{c}_A = -T_{L_A}(c_A, e) + b_A R m_A - u_A c_A - \lambda c_A, \tag{2}$$

$$\dot{p}_A = T_{L_A}(c_A, e) - \lambda p_A. \tag{3}$$

The transcription and translation rates are given by:

$$T_{X_A}(e) = \frac{\omega_A e}{\pi_A + e}, \tag{4}$$

$$T_{L_A}(c_A, e) \quad = \quad \frac{c_A}{n_A}\frac{\gamma_{\max} e}{K_\gamma + e}. \tag{5}$$

The complete derivations for these transcription and translation rates are given in [4]. Table S4 lists the circuit parameter descriptions and their default values.

SN1.2 A model of an *E. coli* host cell

The host model represents a simplified *E. coli* cell with:

1. A simple metabolism where external substrate s_X is imported to become internal substrate s_I and converted into energy e.
2. A coarse-grained proteome with proteins distinguished by their function between transporters p_T, enzymes p_E, ribosomes R (composed of ribosomal protein p_R and rRNA r_R) and other host proteins p_H.
3. Simple gene expression via transcription and translation using mRNA variables m_X and mRNA-ribosome complexes c_X for $x \in \{T, E, R, H\}$.
4. Dynamically calculated growth rate λ as function of the number of actively translating ribosomes.

The complete set of equations for the host model are presented below. Terms in blue demonstrate areas where the host is influenced by the synthetic circuit. Here, we use $x \in \{T, E, R, H\}$ to represent any of the host protein types and $z \in \{T, E, R, H, A\}$ to represent all protein types from both host and circuit. All host parameters are defined in table 5. All variables are diluted by the host growth rate, given by:

$$\lambda = \frac{\gamma_{\max} e}{M(K_\gamma + e)} \sum_z c_z. \tag{6}$$

The circuit model and host model are interconnected through the circuit's consumption of host energy and ribosomes, and calculation of the host's growth rate. The host cell consumes an external substrate molecule s_X. For a complete derivation of all terms in the equations, see [4, 2].

$$\dot{s}_I = v_{\text{imp}}(p_T, s_X) - v_{\text{cat}}(p_E, s_I) - \lambda s_I \tag{7}$$

$$\dot{e} = \phi_e v_{\text{cat}}(p_E, s_I) - \sum_z [n_z T_{L_z}(c_z, e)] - \lambda e \tag{8}$$

$$\dot{m}_x = T_{X_x}(e) + T_{L_x}(c_x, e) - b_x R m_x + u_x c_x - (\lambda + \delta_{m_x}) m_x \tag{9}$$

$$\dot{c}_x = -T_{L_x}(c_x, e) + b_x R m_x - u_x c_x - \lambda c_x \tag{10}$$

$$\dot{p}_{x\setminus R} = T_{L_x}(c_x, e) - (\lambda + \delta_{p_x})p_x \tag{11}$$

$$\dot{p}_R = T_{L_R}(c_r, e) - \beta_r p_R r + \mu_r R - (\delta_{p_R} + \lambda)p_R \tag{12}$$

$$\dot{r} = T_{X_r}(e) - \beta_r p_R r + \mu_r R - (\delta_r + \lambda)r \tag{13}$$

$$\dot{R} = -\sum_z \left[T_{X_z}(c_z, e) - b_z R m_z + u_z c_z\right] + \beta_r p_R r - \mu_r R - (\delta_R + \lambda)R. \tag{14}$$

Import and catabolism obey Michaelis-Menten dynamics, with rates given by:

$$v_{\text{imp}}(p_T, s_X) = \frac{p_T v_T s_X}{k_T + s_X}, \tag{15}$$

$$v_{\text{cat}}(p_e, s_I) = \frac{v_e s_I}{k_e + s_i}. \tag{16}$$

Transcription and translation rates T_{L_x} and T_{L_x} take the same form as those for the synthetic circuit (Eqs. 4,5).

SN1.3 Modelling a population of engineered cells

The host model described above dynamically calculates the cell growth rate λ. This growth rate can be used as a 'birth rate' to model a population of cells via simple ODE model for population size N:

$$\dot{N} = \lambda N. \tag{17}$$

Here, we ignore cell death. To replicate batch conditions, we supply the system with a large quantity of external substrate s_X, which is consumed by all cells within the population:

$$\dot{s}_X = -v_{\text{imp}}(p_T, s_X)N \tag{18}$$

The model is parameterised such that substrate is completely consumed within 4-6 hours, and population size reaches steady state within ≈ 12 hours. A single simulation is run for 24 hours, after which the population size is reset to 1000 cells, and external substrate is replenished.

SN1.4 Modelling mutation and competition between mutant strains

We define a set of n discrete 'mutation states', each differing in its parameterisation (e.g. each with a different value of ω_A). Each mutation state in the model represents a different strain of mutant cells. Each mutation state obeys its own internal and population dynamics. We therefore replicate each of the internal variables n times, one for each mutation state, and model each population size individually. We introduce competition

by updating the external substrate so that it is consumed by all of the mutation states, replicating a physical batch culture consisting of different mutant strains consuming the same nutrient supply:

$$\dot{s}_x = \sum_{j=1}^{n} -v_{\mathrm{imp}_j}(p_{T_j}, s_X)N_j \tag{19}$$

Mutation is modelled by introducing transition rates between different mutation states. If state i mutates into state j at a rate σ_{ij}, then the ODE for the ith population is given by

$$\dot{N}_i = \lambda_i N_i + \sum_{j\neq i} [\sigma_{ji}N_j - \sigma_{ij}N_i] \tag{20}$$

Evolution is then captured through the combined forces of mutation (via rates σ_{ij}) and selection (through differences in growth rates λ_i between competing mutation states).

To define the 'mutation scheme' (i.e. the choices of σ_{ij}), we make use of terminology from [6]. We define the 'dimension' d of a system as the number of parameters which we wish to mutate, and the states per dimension s as the the number of possible values that each parameter can take. For example, suppose we have a circuit consisting of two genes A and B, and we assume that the maximal transcription rates of each gene ω_A and ω_B can mutate, taking one of three values: 50, 20 or 0 mc min^{-1}. Then this system has a dimension $d = 2$ and states per dimension $s = 3$. In this example, the total number of mutation states is 2^3, where each mutation state represents a unique pair of values of ω_A and ω_B. In general, the number of mutation states for a system is d^s. While it is possible to mutate various circuit parameters, increasing d significantly increases computational costs. For this reason and for simplicity, we assume throughout that mutations only affect promoters, choosing only maximal circuit transcription rates ω_z as mutatable parameters. Promoters have been shown to be the genetic parts most vulnerable to loss-of-function mutations [3]. We also assume that mutation states are evenly spaced in function so that e.g. for $s = 5$ and $d = 1$, the 5 states represent 100%, 75%, 50%, 25% and 0% of the designed level of ω_A. It has been demonstrated that some results can only be explained with the help of mutation heterogeneity (i.e. $s > 2$) [6, 7]. However, having a large number of states per dimension can significantly increase model complexity and computation time without significantly altering results. For this reason, we assume $s = 4$ for all systems throughout the paper.

To fully define the mutation scheme, we must define the transition rates σ_{ij} between each pair of mutation states. For a system with $n = 4^d$ mutation states, we can present these transition rates as an $n \times n$ matrix. For simplicity, we make four key assumptions:

1. Mutations only affect a single gene at a time.

2. Mutations are assumed to be point mutations (large repeated sequences are assumed to already have

been eliminated).

3. The more extreme a mutation, the less likely it is to occur.

4. Only mutations which inhibit function can occur.

We use a single parameter $\sigma = 10^{-6}$ min^{-1} which represents the rate of mutation between 'adjacent' states (i.e. the smallest mutation distance). The value of this parameter was chosen to be small enough that the spread of mutation states comes primarily from their outcompetition of other states rather than from the emergence of new mutations, while not being so small as to yield excessively large lifespans. A visual representation of the mutation schemes for $d = 1, 2, 3$ are presented in figure 1. Matrices for $d = 1$ and 2 are presented in tables 6 and 7. The matrix for $d = 3$ follows the same form.

The combined model captures circuit dynamics, host-circuit interactions, population growth and mutation, and leads to open-loop performance which falls over time, consistent with previous models and experimental data [6, 8] (Fig. 2).

SN1.5 Modelling control strategies

Having defined the core model for a simple synthetic circuit consisting of a single gene A, we now augment this model to consider eleven different circuits, representing different controller topologies. Table S1 outlines the name, control input and mechanism of each one. Circuit parameters for the process are the same as those for the open-loop system (Supplementary Note 4). Additional controller parameters are presented in Table S8. Here we represent different synthetic gene types using the following labels: A: Process output, B: Transcription factor, C: sRNA, D: Synthetic enzyme. All synthetic proteins impact the host model in the same way as the process protein outlined in Supplementary Note SN1.2.

CLp_ATX This circuit produces a transcription factor p_B from the same gene as the process output p_A and inhibits the shared gene (Fig. 4a). Control is enacted via a regulatory Hill function which affects the transcription rate of the gene, so that output is more restricted when there are large amounts of p_B (corresponding to large amounts of p_A):

$$\Theta(p_B) = \frac{k_B^2}{p_B^2 + k_B^2} \tag{21}$$

The full set of equations are given below. Terms added by the controller are highlighted in blue. In line with the host model and other established models which do not account for polysome formation, we assume that each transcript m_A can be bound by a single ribosome at either of two ribosome binding sites to form the

corresponding complex c_A or c_B [4, 9, 6, 10].

$$\dot{m}_A = T_{X_A}(e)\Theta(p_B) + T_{L_A}(c_A, e) + T_{L_B}(c_B, e) \\ - b_A R m_A + u_A c_A - b_B R m_B + u_B c_B - (\lambda + \delta_{m_A}) m_A, \tag{22}$$

$$\dot{c}_A = -T_{L_A}(c_A, e) + b_A R m_A - u_A c_A - \lambda c_A, \tag{23}$$

$$\dot{p}_A = T_{L_A}(c_A, e) - \lambda p_A, \tag{24}$$

$$\dot{c}_B = -T_{L_B}(c_B, e) + b_B R m_A - u_B c_B - \lambda c_B, \tag{25}$$

$$\dot{p}_B = T_{L_B}(c_B, e) - \lambda p_B. \tag{26}$$

CLp_ATL This circuit produces a controller protein p_B from the same gene as the process output p_A. This controller protein acts as an activatory transcription factor for a second gene C which produces sRNA molecules, via the regulatory Hill function:

$$\Phi(p_B) = \frac{p_B^2}{p_B^2 + k_B^2} \tag{27}$$

Control is enacted through the sequestration of mRNA by sRNA so that output is more restricted when there are large amounts of synthetic protein (Fig. 4b), at a rate a_C:

$$\dot{m}_A = T_{X_A}(e) + T_{L_A}(c_A, e) + T_{L_B}(c_B, e) \\ - b_A R m_A + u_A c_A - b_B R m_B + u_B c_B - a_C m_A r_C - (\lambda + \delta_{m_A}) m_A, \tag{28}$$

$$\dot{c}_A = -T_{L_A}(c_A, e) + b_A R m_A - u_A c_A - \lambda c_A, \tag{29}$$

$$\dot{p}_A = T_{L_A}(c_A, e) - \lambda p_A, \tag{30}$$

$$\dot{c}_B = -T_{L_B}(c_B, e) + b_B R m_B - u_B c_B - \lambda c_B, \tag{31}$$

$$\dot{p}_B = T_{L_B}(c_B, e) - \lambda p_B, \tag{32}$$

$$\dot{r}_C = T_{X_C}(e)\Phi(p_B) - a_C m_A r_C - (\lambda + \delta_{r_C}) r_C. \tag{33}$$

CLλTX This circuit exploits a promoter that is sensitive to host growth rate λ to produce a transcription factor p_B from gene B which inhibits the process gene A (Fig. S16a). We employ a regulatory Hill function so that more controller proteins are produced when the cell is stressed (i.e. low growth):

$$\Theta(\lambda) = \frac{k_\lambda^2}{\lambda^2 + k_\lambda^2} \tag{34}$$

This controller protein inhibits the process gene A via the regulatory function:

$$\Theta(p_B) = \frac{k_B^2}{p_B^2 + k_B^2} \tag{35}$$

This means that process expression is increased when growth is high and reduced when growth is low. This system is modelled as follows:

$$\dot{m}_A = T_{X_A}(e)\Theta(p_B) + T_{L_A}(c_A, e) - b_A R m_A + u_A c_A - (\lambda + \delta_{m_A}) m_A, \tag{36}$$

$$\dot{c}_A = -T_{L_A}(c_A, e) + b_A R m_A - u_A c_A - \lambda c_A, \tag{37}$$

$$\dot{p}_A = T_{L_A}(c_A, e) - \lambda p_A, \tag{38}$$

$$\dot{m}_B = T_{X_B}(e)\Theta(\lambda) + T_{L_B}(c_B, e) - b_B R m_B + u_B c_B - (\lambda + \delta_{m_B}) m_B, \tag{39}$$

$$\dot{c}_B = -T_{L_B}(c_B, e) + b_B R m_B - u_B c_B - \lambda c_B, \tag{40}$$

$$\dot{p}_B = T_{L_B}(c_B, e) - \lambda p_B. \tag{41}$$

CLλTL This circuit exploits a promoter that is sensitive to host growth rate λ to produce a transcription factor p_B from gene B. We use a regulatory Hill function so that more controller proteins are produced when the cell is stressed (i.e. low growth):

$$\Theta(\lambda) = \frac{k_\lambda^2}{\lambda^2 + k_\lambda^2} \tag{42}$$

This controller protein acts as an activatory transcription factor on a gene C which produces sRNA molecules, via the regulatory function:

$$\Phi(p_B) = \frac{p_B^2}{p_B^2 + k_B^2} \tag{43}$$

Control of the process is then enacted through sRNA combining with and deactivating mRNA of the process gene at a rate a_C (Fig. 6a). The system is modelled as follows:

$$\dot{m}_A = T_{X_A}(e) + T_{L_A}(c_A, e) - b_A R m_A + u_A c_A - a_C m_A r_C - (\lambda + \delta_{m_A}) m_A, \tag{44}$$

$$\dot{c}_A = -T_{L_A}(c_A, e) + b_A R m_A - u_A c_A - \lambda c_A, \tag{45}$$

$$\dot{p}_A = T_{L_A}(c_A, e) - \lambda p_A, \tag{46}$$

$$\dot{m}_B = T_{X_B}(e)\Theta(\lambda) + T_{L_B}(c_B, e) - b_B R m_B + u_B c_B - (\lambda + \delta_{m_B}) m_B, \tag{47}$$

$$\dot{c}_B = -T_{L_B}(c_B, e) + b_B R m_B - u_B c_B - \lambda c_B, \tag{48}$$

$$\dot{p}_B = T_{L_B}(c_B, e) - \lambda p_B, \tag{49}$$

$$\dot{r}_C = T_{X_C}\Phi(p_B) - a_C m_A r_C - (\lambda + \delta_{r_C}) r_C. \tag{50}$$

CLλprotein-free Just like CLλTL, this controller exploits growth-sensitive promoters and enacts control via sRNA. Here, however, the growth-sensitive gene produces the sRNA directly (Fig. S21a). This approach is 'burden-free' in the sense that it does not require the production of any additional proteins. The disadvantage of this approach is the lack of practical tuneability or amplification, as it relies on existing promoters. It makes use of a regulatory function so that more sRNA is produced at low growth:

$$\Theta(\lambda) = \frac{k_\lambda^2}{\lambda^2 + k_\lambda^2} \tag{51}$$

The model is defined as follows:

$$\dot{m}_A = T_{X_A}(e) + T_{L_A}(c_A, e) - b_A R m_A + u_A c_A - a_C m_A r_C - (\lambda + \delta_{m_A}) m_A, \tag{52}$$

$$\dot{c}_A = -T_{L_A}(c_A, e) + b_A R m_A - u_A c_A - \lambda c_A, \tag{53}$$

$$\dot{p}_A = T_{L_A}(c_A, e) - \lambda p_A, \tag{54}$$

$$\dot{r}_C = T_{X_C}\Theta(\lambda) - a_C m_A r_C - (\lambda + \delta_{r_C}) r_C. \tag{55}$$

CLQSTX This controller uses quorum sensing to enable cells to communicate and sense the population-wide process output. An enzyme p_D is produced from the same gene as p_A. This enzyme produces the internal metabolite i_D from internal substrate s_I at a rate:

$$v_{\text{cat}_D}(s_I, p_B) = \frac{p_D v_{\max D} s_I}{k_D + s_I} \tag{56}$$

i_D can exit the cell to become external metabolite x_D. (Note that x_D represents a single variable for the entire population, where other variables are tracked on a per-cell basis.) The rate at which i_D is exported is proportional to the difference in concentration inside vs outside the cell, given by:

$$\Psi(i_D, x_D) = v_{\text{exp}_D}\left(\frac{i_D}{V_{\text{cell}}} - \frac{x_D}{V_{\text{culture}}}\right), \tag{57}$$

where V_{cell} and $V_{culture}$ represent the volumes of the cell and the culture. Another gene B constitutively produces transcription factors p_B, which become functional by binding with i_D at a rate b_{BD}. The functional transcription factor $[BD]$ inhibits the production of p_A and p_D, completing the feedback loop (Fig. S3a).

This is enacted by the regulatory function:

$$\Theta([BD]) = \frac{k_{BD}^2}{[BD]^2 + k_{BD}^2}.$$

The full model is given below:

$$\dot{m}_A = T_{X_A}(e)\Theta(BD) + T_{L_A}(c_A, e) + T_{L_D}(c_D, e) \\ - b_A R m_A + u_A c_A - b_D R m_D + u_D c_D - (\lambda + \delta_{m_A}) m_A, \tag{58}$$

$$\dot{c}_A = -T_{L_A}(c_A, e) + b_A R m_A - u_A c_A - \lambda c_A, \tag{59}$$

$$\dot{p}_A = T_{L_A}(c_A, e) - \lambda p_A, \tag{60}$$

$$\dot{m}_B = T_{X_B}(e) + T_{L_B}(c_B, e) - b_B R m_B + u_B c_B, \tag{61}$$

$$\dot{c}_B = b_B R m_B - u_B c_B - T_{L_B}(c_B, e) - \lambda c_B, \tag{62}$$

$$\dot{p}_B = T_{X_B}(e) - b_{BD} i_D p_B + u_{BD}[BD] - \lambda p_B, \tag{63}$$

$$\dot{c}_D = b_D R m_A - u_D c_D - T_{L_D}(c_D, e) - \lambda c_D, \tag{64}$$

$$\dot{p}_D = T_{L_D}(c_D, e) - \lambda p_B \tag{65}$$

$$\dot{i}_D = v_{\text{cat}_D}(s_I, p_B) - \Psi(i_D, x_D) - b_{BD} i_D p_B + u_{BD}[BD] - \lambda i_D, \tag{66}$$

$$\dot{x}_B = \sum_{j=1}^{n} \Psi_j(i_D, x_D) N_j, \tag{67}$$

$$[\dot{BD}] = b_{BD} i_D p_B - u_{BD}[BD] - \lambda [BD] \tag{68}$$

We have not included a model for a system that uses quorum sensing and implements control using sRNA (CLQSTL). This is because (a) population-based control is demonstrated to be less effective than growth-based control (Supplementary Note SN2) and (b) the computational cost to run simulations with such a model becomes excessively large.

Multi-input controllers We considered five combined controllers, called CLp_ATXλTL, CLp_ATLλTX, CLp_ATLλTL CLp_ATXλTX and CLp_ATLλPF (PF ≔ protein-free). These use the same control inputs and mechanisms as previous systems, but combine them together (Fig. 8a-c). There are no new parameters or variables. In each system, a transcription factor p_{B1} is produced from the same gene as the process output A to enact protein-based control. With the exception of CLp_ATLλPF, a second transcription factor p_{B2} is produced from a growth-sensitive promoter to enact growth-based control. Each of p_{B1} and p_{B2} implement control by either preventing transcription (by inhibiting the process gene A) or preventing translation (by

activating sRNA r_C which binds with process mRNA m_A). These are modelled as follows, where terms unique to one of the three implementations are highlighted, and terms in brown (representing gene $B2$) are not included in the CLp_ATLλPF model:

$$\dot{m}_A = T_{X_A}(e)\underbrace{\Theta(p_{B1})}_{\text{CL}p_A\text{TX}\lambda\text{TL}} \cdot \underbrace{\Theta(p_{B2})}_{\text{CL}p_A\text{TL}\lambda\text{TX}} \cdot \underbrace{\Theta(p_{B1})\Theta(p_{B2})}_{\text{CL}p_A\text{TX}\lambda\text{TX}} + T_{L_A}(c_A, e) + T_{L_{B1}}(c_{B1}, e)$$

$$- b_A R m_A + u_A c_A - b_{B1} R m_{B1} + u_{B1} c_{B1} - a_C m_A r_C - (\lambda + \delta_{m_A}) m_A, \tag{69}$$

$$\dot{c}_A = -T_{L_A}(c_A, e) + b_A R m_A - u_A c_A - \lambda c_A, \tag{70}$$

$$\dot{p}_A = T_{L_A}(c_A, e) - \lambda p_A, \tag{71}$$

$$\dot{c}_{B_1} = -T_{L_{B1}}(c_{B1}, e) + b_{B1} R m_{B1} - u_{B1} c_{B1} - \lambda c_{B1}, \tag{72}$$

$$\dot{p}_{B_1} = T_{L_{B1}}(c_{B1}, e) - \lambda p_{B1}, \tag{73}$$

$$\dot{m}_{B_2} = T_{X_{B2}}(e)\Theta(\lambda) + T_{L_{B2}}(c_{B2}, e) - b_{B2} R m_{B2} + u_{B2} c_{B2} - (\lambda + \delta_{m_{B2}}) m_{B2}, \tag{74}$$

$$\dot{c}_{B_2} = -T_{L_{B2}}(c_{B2}, e) + b_{B2} R m_{B2} - u_{B2} c_{B2} - \lambda c_{B2}, \tag{75}$$

$$\dot{p}_{B_2} = T_{L_{B2}}(c_{B2}, e) - \lambda p_{B2}, \tag{76}$$

$$\dot{r}_C = T_{X_A}(e)\underbrace{\Phi(p_{B1})}_{\text{CL}p_A\text{TX}\lambda\text{TL}} \cdot \underbrace{\Phi(p_{B2})}_{\text{CL}p_A\text{TL}\lambda\text{TX}} \cdot \underbrace{\Phi(p_{B1})\Phi(p_{B2})}_{\text{CL}p_A\text{TL}\lambda\text{TL}} \cdot \underbrace{\Theta(\lambda)}_{\text{CL}p_A\text{TL}\lambda\text{PF}}$$

$$- a_C m_A r_C - (\lambda + \delta_{r_C}) r_C. \tag{77}$$

SN1.6 Parameter boundary values for optimisations

Above, we have defined the models for the different controllers considered throughout. We performed multi-objective optimisations on these models as described in Methods 4.3, with the parameterisations defined in Table S8. Here, we detail our choices for the upper and lower bounds of each parameter varied in the optimisations. In general, we aimed to set bounds that represented wide but biologically feasible ranges.

First, we consider the maximal transcription rate parameters ω_A, ω_B, ω_C and ω_D. For the open-loop system, we set the upper bound of ω_A to be beyond the point where cells are so burdened that further increasing transcription damages output. We set the lower bound to be a point where the difference in growth rates between functional and non-functional cells was negligible, and therefore that the dominant cause of mutant spread was the emergence of new mutants, rather than natural selection through differences in growth rate. $[10^{-1}\ \ 10^3]$ mc min^{-1} satisfied these conditions. For the other maximal transcription rate parameters (ω_B, ω_C and ω_D), we used the same range.

Next, we consider the controller threshold parameters for the phenomenological models (k_A, k_λ, k_P), which determine the strength of control. Note that increased control strength is represented by increased values of

k_A and k_P, but reduced values of k_λ, because high burden corresponds to high output and low growth. In each case, we set the 'lower bound of control strength' to be a value which represents an ineffective controller which has negligible impact on the initial output P_0. The 'upper bound of control strength' was determined to be within 1-2 orders of magnitude of the corresponding quantity. (E.g. for p_A within a typical range of 10^4 to 10^5 molecules per cell (Fig. 2e), 10^3 mc represents a reasonable lower bound for k_A.) This yielded the following ranges for parameters k_A, k_λ and k_P: $[10^3 \;\; 10^6]$ mc, $[10^{-6} \;\; 10^{-1}]$ min^{-1}, $[10^7 \;\; 10^{10}]$ mc. For the mechanistic models, k_λ is equivalent to k_λ in the phenomenological model, so we use the same range. Transcription factors p_B and $[BD]$ are of the same order of magnitude as p_A, so for each we use the range $[10^3 \;\; 10^6]$ mc.

Finally, we consider binding rate parameters b_B and b_D. Here, we take values aligned with those used in a previous analysis by Nikolados et al. [1]. In this work, the authors consider a synthetic circuit which describes an inducible gene. They vary the binding rate parameter for this gene between 10^{-2} and 10^0 mc min^{-1}. We therefore chose $b_A = 10^{-1}$ mc min^{-1} as a sensible binding rate for our open-loop system. The overall ribosome binding strength of a gene X in this model is a function of both the binding rate b_X and the unbinding rate u_X. In [1], the authors also vary u_X to achieve weaker ribosome binding strengths. For simplicity, we chose not to vary the u_B and u_D parameters in our optimisations, but instead considered a wider range of b_B and b_D to achieve lower ribosome binding strengths. $[10^{-3} \;\; 10^0]$ mc min^{-1} therefore represents a sensible range for b_B and b_D.

SN2 Supplementary Note 2: Population-based control shows poor performance even with optimised parameters

Controllers which use quorum-sensing have become widely used in synthetic biology due to their ability to sense properties of a system across an entire population [11]. Here we consider whether a quorum-sensing-based controller could be effective at improving the evolutionary performance of a synthetic circuit. The circuit is designed so that a single cell can sense synthetic circuit activity at the population level, and control its own level of synthetic protein production in response. For the sake of reducing computational complexity, we consider actuation via a transcription factor (CLQSTX) and compare this against CLλTX (Fig. S3a). We assume that the burden of the controller is purely translational; host energy/ribosomes are used to produce the synthetic enzymes, but precursors aren't consumed in the subsequent production of quorum sensing molecules. The real performance of such a controller could therefore be worse due to this additional metabolic burden.

Theoretically, intra-circuit and growth-based controllers function by allowing mutated cells to respond to a reduction in their base transcription rate by alleviating control and pushing transcription back up. This controller, on the other hand, relies on unmutated cells, which sense when the population-level output is decreasing and attempt to compensate for the rise of mutants with increased production of output p_A. While $CLQSTX$ and CLλTX have comparable short-term performance ($\tau_{\pm10}$), the quorum-sensing-based controller cannot match the growth-based controller in the long-term (τ_{50}) (Fig. S3b-g). For systems of the same initial output, CLQSTX sees a larger spike in its growth and protein production in response to the daily replenishing of substrate, resulting in a reduced growth rate, and giving a greater selective advantage to mutants with non-functional processes (Fig. S6a,b,d). However, when the controller mutates, the protein produced per cell increases significantly more than for CLλTX (Fig. S6c). Despite such mutants having significantly reduced growth, it is their arrival in the short-term before being outcompeted that enables them CLQSTX to compete with CLλTX in the short term (Fig. S6e). As with other systems, control strength should be maximised (k_B minimised). Transcription of the transcription factor (ω_B) and ribosome binding rate of the enzyme (b_D) show clear optimal values across all samples on the Pareto front, with process transcription (ω_A) and transcription factor binding rate (b_D) varying across the front (Fig. S4).

Robustness of this controller is far worse than CLp_ATX. Compared with CLλTX, while τ_{50} is more robust to parametric variation, its initial output P_0 shows reduced robustness, and fewer controllers maintain $\tau_{\pm10} = \tau_{90}$. (13.5% (23.7%) of controllers fail when parameters are varied by ±10% (±25%) compared with 11.1% (16.8%) for CLλTX). When parameters are varied by up to ±10% (±25%), standard deviations in P_0, $\tau_{\pm10}$, τ_{50} are 12.3% (31.4%), 21.8% (33.2%) and 5.1% (14.3%) (compared with 9.1% (22.6%), 18.1%

(43.9%) and 9.2% (17.35%) for CLλTX) (Fig. S3h-j). A comprehensive robustness analysis is provided in Supplementary Note SN3.

SN3 Supplementary Note 3: Robustness analyses for all control systems

In this work, we have analysed the robustness of eleven controllers (outlined in Table S1) by evaluating standard deviations in the percentage changes in P_0, $\tau_{\pm 10}$ and τ_{50} when optimal parameters were randomly varied by ±10% and ±25%. Here, we present a further analysis which also accounts for the additional metrics: τ_{90} and P_{max} (defined in Methods 4.4). In addition, we evaluate how the optimal Pareto fronts themselves vary under parameter variation, noting that individual designs may show significant change in their output while remaining close to the front when parameters are randomly varied. Robustness metrics for each controller are summarised in Table S2, with means and standard deviations given.

For intra-circuit control, 100% of optimal designs retain $\tau_{\pm 10} = \tau_{90}$ upon parameter variation. For CLp_ATX, both with and without mutation of the k_B parameter, $P_{max} = P_0$, suggesting that total protein concentration never exceeds its initial value. For CLp_ATL, there are a handful of designs for which $P_{max} \neq P_0$, but the difference in robustness between P_{max} and P_0 is negligible. In this case, therefore, P_{max} and τ_{90} do not provide much additional insight. Across intra-circuit controllers, initial output P_0 is most sensitive to parametric variation, while τ_{50} shows the smallest variation (Table S2). For CLp_ATX, as the initial output P_0 of optimal designs increases, output metrics P_0 and P_{max} become less sensitive to parametric variation, while longevity metrics $\tau_{\pm 10}$, τ_{50} and τ_{90} become more sensitive to parametric variation (Fig. 4g-i, S8a-b). For CLp_ATL, the same trend is observed in longevity metrics $\tau_{\pm 10}$, τ_{50} and τ_{90}, but percentage changes in output metrics P_0 and P_{max} are more consistent across the front (Fig. 4j-l, S8i-j). While P_0, $\tau_{\pm 10}$ and τ_{50} can individually vary by more than 25% when parameters are varied by ±25%, randomised controllers remain very close to the Pareto front, with only CLp_ATL showing a small reduction in performance for a few designs with very low initial output P_0 (Fig. S8c-d,g-h,k-l).

For growth-based control, P_{max} is more robust to parametric variation than P_0. τ_{90} is more robust than $\tau_{\pm 10}$, reflecting the fact that $\tau_{\pm 10}$ can vary significantly if parameter variation causes $\tau_{\pm 10}$ to be different from τ_{90}. For CLλTX, P_{max} is the most robust metric to parametric change. However, for the sRNA-mediated systems (CLλTL and CLλp-free), τ_{50} is the more robust metric (Table S2). This suggests that precisely controlling the output is more challenging for systems with sRNA-mediated control. For CLλTX, variation in P_0 and P_{max} does not change significantly as original initial output P_0 is increased, but variation in τ_{90} and τ_{50} is larger at higher values of P_0 (Fig. S16h-j, S19a-b). For CLλTL, this increase in τ_{90} and τ_{50} is also observed, but unlike CLλTX, P_0 and P_{max} also show more variability at larger initial values of P_0 (Fig. 6f-h, S19e-f). Out of all controllers considered, CLλTL has the largest number of designs which fail to maintain $\tau_{\pm 10} = \tau_{90}$ (21.5% (28.2%) when parameters are varied by ±10% (±25%)). CLλp-free has two different design paradigms for low and high initial output P_0. In the high output regime, where performance

is poor, each metric is much more robust than in the low output regime with greater performance (Table S2, Fig. S21h-j, S5a-b). For all growth-based controllers, despite potentially large variations in individual metrics, designs do not fall significantly off the Pareto front when considering only P_0 and τ_{50}. However, each controller sees a large number of designs falling off the Pareto front when comparing P_0 and $\tau_{\pm 10}$ (Fig. S19c-d,g-h, S5c-d). Although it shows the best performance, this drop from the Pareto front is largest and most widespread for CLλTL.

For CLQSTX, P_{max} is more robust to parametric variation than P_0 and τ_{90} is more robust than $\tau_{\pm 10}$. τ_{50} is the metric most robust to parametric variation, while P_{max} and τ_{90} both show similar levels of robustness (Table S2). As the original initial output P_0 is increased, the variation in each metric does not vary significantly when parameters are varied by $\pm 10\%$. However, when parameters are varied by $\pm 25\%$, the robustness varies more significantly across different original designs (Fig. S3h-j, S5e-f). When considering only P_0 and τ_{50}, designs do not appear to fall significantly off the Pareto front, despite them potentially showing significant variation in P_0 and τ_{50} individually. However, when comparing P_0 and $\tau_{\pm 10}$, a large number of designs fall off the Pareto front, with a wide and unpredictable spread of outputs (Fig. S5g-h).

Next we consider the four multi-input controllers: CLp_ATXλTL, CLp_ATLλTX, CLp_ATLλTL and CLp_ATXλTX. For intra-circuit control and growth-based control, there is a trade-off between performance and robustness, with sRNA-mediated feedback typically yielding better performance and reduced robustness compared with feedback mediated by transcription factors. However, CLp_ATXλTX offers no improvement in robustness compared with the other multi-input controllers despite a reduction in performance (Table S2, Fig. S24i-k, S29a-b). Overall, CLp_ATLλTX and CLp_ATLλTL do not differ significantly in their robustness. However, CLp_ATXλTL is notably worse than these two, particularly in the P_0 and P_{max} metrics (Fig. 8h-j, S26, S28a-b,e-f,i-j). Further, CLp_ATXλTL has more designs which fail to maintain $\tau_{\pm 10} = \tau_{90}$. This suggests that multi-input control should be designed with an sRNA-mediated intra-circuit component. However, it is worth noting that the variability in P_0, P_{max} and τ_{90} is reduced for original designs CLp_ATXλTL with high initial output P_0, suggesting that it could still be effective in systems with a higher level of burden (Fig. S26a, S28a-b). For each of these controllers, τ_{50} is the metric most robust to parametric variation. When parameters are randomly varied, the relationship between P_0 and τ_{50} does not significantly change across the Pareto front. However, there is a set of designs for each controller where the relationship between P_0 and $\tau_{\pm 10}$ falls significantly off the front. CLp_ATXλTX and CLp_ATXλTL show the largest and most widespread drop from the front, while the drop is least steep and widely varied for CLp_ATLλTX (Fig. S28c-d,g-h,k-l, S29c-d).

As with CLλp-free, CLp_ATLλPF has two different parameter regimes for low and high initial output P_0 (Fig. S27). However, whereas CLλp-free displays poor robustness in the low output regime, CLp_ATLλPF is

able to maintain very good robustness across the front (Fig. S24l-n, S29e-f). In particular, 100% of designs retain $\tau_{\pm 10} = \tau_{90}$. This also means that the robustness of the additional metrics P_{max} and τ_{90} are very similar or exactly the same as the robustness of P_0 and $\tau_{\pm 10}$. Designs remain very close to the original Pareto front after parameter variation (Fig. S29g-h).

For every controller, the mean percentage change in P_0 is positive, while the mean percentage change in $\tau_{\pm 10}$ is negative (Table S2). This suggests that maximising $\tau_{\pm 10}$ is the most challenging objective to achieve, as parameter variation is more likely to reduce this objective.

SN4 Supplementary Note 4: Analysing the design space of intra-circuit and growth-based controllers

The objective of this work is to employ a theoretical modelling framework to inform design choices for the *in vivo* implementation of genetic controllers which extend the lifespans of genetic circuits. In the main text, we focused our analysis on multi-objective optimisations for each controller topology to determine differences in their performance (through the optimisation outputs), and how to design them (through the optimal parameterisations). We also evaluated the robustness of the different controller topologies to parametric variation through a Monte Carlo analysis. This is important to consider when it comes to *in vivo* implementation as optimal parameterisations cannot be achieved precisely as a result of limitations in part selection and biological constraints/noise.

Here, we take a more detailed look at some of the key design choices that influence evolutionary longevity, to better understand the relative importance of different parameters, and to evaluate the size of the design space which improves $\tau_{\pm 10}$ and τ_{50}. To do this, we consider a nominal open-loop process with maximal process transcription rate $\omega_A = 50$ mc min^{-1}. We then perform parameter sweeps on controllers which influence this process and evaluate the importance of the different key parameters. This will give an indication of the ease with which performance improvements can be obtained, given that parameters can't necessarily be fixed at their optimum.

First, we consider CLp_ATX. This controller has two key design parameters: the threshold parameter k_B, which determines the 'strength' of control (i.e. its sensitivity to changes in output p_A and subsequent influence on expression) and the ribosome binding rate b_B, which determines the abundance of transcription factors. For the nominal open-loop process, we performed a parameter sweep on these two parameters, and compared the resulting designs against open-loop systems of equal initial outputs P_0. The initial output P_0 increases as we reduce the control strength (increase k_B) and reduce the ribosome binding rate (decrease b_B) (Fig. S9a). For systems of equal initial output (contours in Fig. S9), those with stronger control and reduced binding strength show the best performance (Fig. S9b). While improvements to both $\tau_{\pm 10}$ and τ_{50} are possible, a large portion of the design space yielded systems which performed worse than open-loop. $\tau_{\pm 10}$ can be improved by a more significant margin than τ_{50}, and across a wider portion of the design space, suggesting that it is easier to boost short-term performance than long-term performance (Fig. S9c-d).

Next, we considered CLp_ATL. This controller also uses the parameters k_B and b_B, again defining control strength and transcription factor abundance. In addition, this controller relies on the maximal transcription rate parameter ω_C, which determines the expression of sRNA. The higher the value of this parameter, the fewer transcription factors are needed to achieve the same level of inhibition, so this parameter also influences

the strength of the controller. We fixed the maximal transcription rate of sRNA ω_C at its maximum value of $\omega_C = 1000$ mc min^{-1}, in line with optimal designs. We then considered the trade-off between controller protein expression and control strength by varing b_B and k_B (Fig. S13). Given systems of equal initial output (contours), those which perform best are those with stronger control (low k_B) and lower expression (low b_B). The portion of the design space which yields an improvement versus open-loop is much larger than that of CLp_ATX, suggesting that, even though CLp_ATL is less robust as a topology, it is still easier to design systems which improve both $\tau_{\pm 10}$ and τ_{50}. We also repeated this analysis, varying b_B and ω_C for fixed $k_B = 10^3$ mc (Fig. S14a-d) and $k_B = 10^4$ mc (Fig. S14e-h). We again see that $\tau_{\pm 10}$ improves across a wider portion of the design space than τ_{50}, and that the best performing designs maximise strength (high ω_C) and minimise expression (low k_B). The design space where both $\tau_{\pm 10}$ and τ_{50} improve is larger when k_B is lower (i.e. control is stronger).

Finally, we considered the growth-based controller topology CLλTX. This system has four key parameters: k_B and k_λ, threshold parameters which determine the strength of control, and ω_B and b_B, the maximal transcription rate and ribosome binding rate of the transcription factor which determine the abundance of controller proteins. We performed two parameter sweeps, firstly on k_B and b_B (Fig. S20a-d) and secondly on k_λ and ω_B (Fig. S20e-h). In each case, we fixed the other parameters at their optimum values. As with the intra-circuit controllers, the maximum possible improvements in $\tau_{\pm 10}$ (almost 300% increase) versus open-loop are greater than those in τ_{50} ($\tilde{1}$50% increase). However, the parameter range where short-term improvements can be achieved by this magnitude is very narrow. In general, design rules for improving τ_{50} align with those for intra-circuit controllers: maximise control strength (via k_B and k_λ) and minimise the abundance of controller proteins (via b_B and ω_B). Notably, the portion of design space which improves $\tau_{\pm 10}$ is smaller than that of τ_{50}. One key reason for this is the existence of parameterisations where $\tau_{\pm 10} \neq \tau_{90}$ (Fig. S20b), as demonstrated in Fig. S18. These results suggest that growth-based controllers are more difficult to engineer in practice for applications which require short-term maintenance of function, but can be particularly beneficial for systems where long-term persistence is more important.

SN5 Supplementary Note 5: Mutating both dissociation constant k_B and maximal transcription rate ω_A of the CLp_ATX controller

Throughout the main text, we assumed that mutation only affects the promoters of circuit genes, as this is the component most prone to mutation [8]. Increasing the number of mutating parameters also significantly increases the computational complexity of the model by increasing the number of mutation dimensions. Here, we present a system which separately mutates the strength of a controller via the dissociation constant k_B.

In section 2.3, we outlined two key factors contributing to the difference in performance between CLp_ATL and CLp_ATX: (1) that CLp_ATL can achieve stronger control using fewer burdensome proteins and (2) the existence of mutations in the controller resulting in strains with increased production. To verify the importance of each of these two factors, we separately considered a transcription-based controller identical to CLp_ATX but where both the maximal process transcription rate ω_A and control strength k_B mutate, thus creating a control system which enables the emergence of mutants with increased output through a reduction in control strength (i.e. factor 2), but without the reduction in burdensome controller proteins (i.e. factor 1).

We optimised this controller using identical objectives and parameter ranges as we did in the main text for CLp_ATX. Whereas non-functional promoters are explicitly defined with $\omega_A = 0$ mc min^{-1}, control strength becomes weaker as k_B gets larger (Eq. 21.) Initial mutation-free simulations suggest that $k_B = 10^6$ mc represents a value where control strength is small enough to have no influence. We therefore model mutation of k_B by setting the fully functional state to have k_B at the designed level, the non-functional state to have $k_B = 10^6$ mc, and intermediate states logarithmically interpolated between these two values. Although this controller outperformed CLp_ATX by a factor of two in the short-term due to the emergence of mutants with broken controllers, it showed minimal improvement in the long-term and was unable to compete with CLp_ATL, demonstrating that the ability to provide stronger control using fewer burdensome proteins is the primary contributor to the enhanced long-term performance of CLp_ATL (Fig. S11a-f). The optimal parameters for this new system align with those for the original CLp_ATX system, demonstrating that this extra dimension of mutation does not qualitatively alter the controller design rules (Fig. S12). The robustness of this controller is very similar to that of the original CLp_ATX system (Fig. S11g-i).

SN6 Supplementary Note 6: Transcriptional burden has negligible effect on controller performance

Our modelling framework, as with others, is based make the assumption that the burden caused by the transcription of genes is insignificant compared with the burden caused by translation [4, 2, 6, 7]. Whilst transcription rates are dependent on the availability of energy e (such that transcription falls as energy falls), the generation of mRNA does not consume this variable (Eq. 8). It is well-documented for *E. coli* that translation dominates the consumption of cellular energy [12, 13, 14], and similar observations are made for other microbes [15]. However, transcriptional burden can have subtle effects on circuit gene expression [16, 17]. Here, we consider the impact of transcriptional burden on CLp_ATX and CLp_ATL by altering Eq. 8 so that the transcription of synthetic genes consumes cellular energy e:

$$\dot{e} = \phi_e v_{\text{cat}}(p_E, s_I) - \phi_{T_X} n_A T_{X_A}(e) - \sum_z [n_z T_{L_z}(c_z, e)] - \lambda e. \tag{78}$$

Here, ϕ_{T_X} defines the energy cost of transcription of a single RNA transcript. We assume that evolution has optimised cellular physiology to minimise the transcriptional burden of host genes.

To evaluate the performance of CLp_ATX with additional transcriptional burden, we re-performed the multi-objective optimisations described in Section 2.3 for a system with $\phi_{T_X} = 1$ (i.e. a high transcriptional burden). We compared the optimal values of $\tau_{\pm 10}$ and τ_{50} against an open-loop system with $\phi_{T_X} = 1$. The front shows equivalent performance to that determined with no transcriptional burden ($\phi_{T_X} = 0$) (Fig. S15a,b). Analysis of the optimal controllers also shows no difference in their designs (Fig. S15c-e).

We evaluated the performance of CLp_ATL controller for a range of ϕ_{T_X} values between 0.0016 and 1 through multi-objective optimisation (comparing performance against an open-loop system with the same value of ϕ_{T_X}, as previously described). As transcriptional burden increases ($\phi_{T_X} \to 1$), performance of the controller falls. At high levels of transcriptional burden, the controller's initial output P_0 is smaller while $\tau_{\pm 10}$ shows a more modest improvement of only up to +200% versus open-loop when compared to low burden systems (Fig. S15f,g). At the highest transcriptional energy consumption, τ_{50} can be worse than open-loop (Fig. S15f,g). As transcriptional burden falls, the performance of the controller converges to the original front (with $\phi_{T_X} = 0$). Optimal parameterisations show very little change, aside from a slight decrease in the maximal transcription rate ω_A of gene A (Fig. S15h-k).

SN7 Supplementary Note 7: Comparing transcriptional and post-transcriptional actuation in growth-based control

In Section 2.3, we demonstrated that, for intra-circuit control, an sRNA-mediated mechanism outperforms a transcription factor mechanism for both $\tau_{\pm 10}$ and τ_{50}, but that this improvement comes at a cost to robustness. In the main text, we focused our growth-based analysis primarily on the sRNA-mediated controller CLλTL. Here, we we report results for the CLλTX controller (Fig. S16a). This is modelled as described in Supplementary Note SN1.5, and we performed multi-objective optimisations in line with the other control systems. Unlike with intra-circuit control, both CLλTX and CLλTL have separate controller genes which can mutate. Therefore, both systems can see the arrival of mutations with higher output. In the 'burden-free' case, (i.e. $n_B = 1$ aa), the performances of CLλTX and CLλTL are therefore very similar (Fig. S16b-g). However, when controller burden is introduced ($n_B = 300, 600$ aa), the importance of being able to provide 'more control for less burden' is increased. Whereas CLλTL is more resistant to increases in controller protein length, the performance of CLλTX worsens significantly, demonstrating that the benefits of the less burdensome post-transcriptional mechanism are still prevalent for growth-based feedback control. Optimal parameters for CLλTX are similar to those which optimise CLλTL, although more controller transcription (higher ω_B) is required to achieve systems of the same initial output (Fig. S17). The robustness of this controller is better than CLλTL when parameters are varied by ±10%, but worse when parameters are varied by ±25%. Standard deviations in P_0, $\tau_{\pm 10}$ and τ_{50} are 9.1% (22.6%), 18.1% (43.9%) and 9.2% (17.35%) when parameters are varied by ±10% (±25%) (Fig. S16h-j). A comprehensive robustness analysis is presented in Supplementary Note SN3 (Fig. S19).

SN8 Supplementary Note 8: Protein-free growth-based control

Here, we consider a protein-free growth-based controller where the growth-sensitive promoters drive sRNA expression directly (rather than via a regulator protein) (Fig. S21a). We call this CLλprotein-free ("Closed-loop, senses λ, no additional proteins"). This topology limits the design space as implementation is restricted to the use of a stress-responsive promoter with specific desirable dynamics but removes the burden associated with regulator protein production. While this system lacks tuneability and flexibility and is outperformed by CLλTL, it always improves $\tau_{\pm 10}$ and τ_{50} versus open-loop (Fig. S21b-g). This circuit therefore presents a cheap and easily implementable way to improve evolutionary longevity, in a similar fashion to the use of autoregulatory transcription factors [18]. This topology suffers from poor robustness in the same manner as CLλTL (Fig. S21h-j). Optimal parameters show differences at high and low initial outputs P_0. At low initial outputs, the strength of control k_λ is consistent with CLλTL, while the maximal transcription rates ω_A and ω_C are reduced. On the other hand, at high initial outputs, ω_A is consistent with CLλTL but control strength is increased (lower k_λ) (Fig. S22). In the low output parameter regime, performance is enhanced, but robustness is significantly worse.

SN9 Supplementary Note 9: Phenomenological modelling of multi-input controllers

To understand the theoretical potential of a control strategy which combines growth-based and intra-circuit feedback, we first considered a phenomenological model where synthetic protein production is inhibited by both the production of p_A and activated by increased growth λ (Fig. S23a). Transcription of the process gene is scaled by the regulatory function:

$$\Psi_A(p_A, \lambda) = \frac{{k_A}^2}{{k_A}^2 + p_A^2} \frac{\lambda^2}{{k_\lambda}^2 + \lambda^2} \tag{79}$$

Multi-objective optimisation of this model reveals that this multi-input controller has the potential to enhance evolutionary longevity significantly beyond what is possible from either type individually (Fig. S23b-d). It is capable of combining the benefits of both intra-circuit feedback, which reduces the growth advantage of intermediate states, and growth-based feedback, which improves the growth rate of the fully functional state (Fig. S23e-f).

Supplementary References

[1] E.-M. Nikolados, A. Y. Weiße, F. Ceroni, and D. A. Oyarzún, "Growth defects and loss-of-function in synthetic gene circuits," *ACS synthetic biology*, vol. 8, no. 6, pp. 1231–1240, 2019.

[2] A. P. Darlington, J. Kim, J. I. Jiménez, and D. G. Bates, "Dynamic allocation of orthogonal ribosomes facilitates uncoupling of co-expressed genes," *Nature communications*, vol. 9, no. 1, p. 695, 2018.

[3] S. C. Sleight and H. M. Sauro, "Visualization of evolutionary stability dynamics and competitive fitness of escherichia coli engineered with randomized multigene circuits," *ACS synthetic biology*, vol. 2, no. 9, pp. 519–528, 2013.

[4] A. Y. Weiße, D. A. Oyarzún, V. Danos, and P. S. Swain, "Mechanistic links between cellular trade-offs, gene expression, and growth," *Proceedings of the National Academy of Sciences*, vol. 112, no. 9, pp. E1038–E1047, 2015.

[5] S. Marino, I. B. Hogue, C. J. Ray, and D. E. Kirschner, "A methodology for performing global uncertainty and sensitivity analysis in systems biology," *Journal of theoretical biology*, vol. 254, no. 1, pp. 178–196, 2008.

[6] D. Ingram and G.-B. Stan, "Modelling genetic stability in engineered cell populations," *nature communications*, vol. 14, no. 1, p. 3471, 2023.

[7] D. P. Byrom and A. P. Darlington, "On the implications of controller resource consumption for the long-term performance of synthetic gene circuits," in *2023 62nd IEEE Conference on Decision and Control (CDC)*, pp. 8844–8850, IEEE, 2023.

[8] S. C. Sleight, B. A. Bartley, J. A. Lieviant, and H. M. Sauro, "Designing and engineering evolutionary robust genetic circuits," *Journal of biological engineering*, vol. 4, pp. 1–20, 2010.

[9] K. Sechkar and H. Steel, "Model-guided gene circuit design for engineering genetically stable cell populations in diverse applications," *Journal of the Royal Society Interface*, vol. 22, no. 223, p. 20240602, 2025.

[10] C. Liao, A. E. Blanchard, and T. Lu, "An integrative circuit–host modelling framework for predicting synthetic gene network behaviours," *Nature microbiology*, vol. 2, no. 12, pp. 1658–1666, 2017.

[11] A. Boo, R. L. Amaro, and G.-B. Stan, "Quorum sensing in synthetic biology: A review," *Current Opinion in Systems Biology*, vol. 28, p. 100378, 2021.

[12] O. Maaløe, "Regulation of the protein-synthesizing machinery—ribosomes, trna, factors, and so on," in *Biological Regulation and Development: Gene Expression*, pp. 487–542, Springer, 1979.

[13] H. Bremer, P. P. Dennis, *et al.*, "Modulation of chemical composition and other parameters of the cell by growth rate," *Escherichia coli and Salmonella: cellular and molecular biology*, vol. 2, no. 2, pp. 1553–69, 1996.

[14] A. Wagner, "Energy constraints on the evolution of gene expression," *Molecular biology and evolution*, vol. 22, no. 6, pp. 1365–1374, 2005.

[15] M. Kafri, E. Metzl-Raz, G. Jona, and N. Barkai, "The cost of protein production," *Cell reports*, vol. 14, no. 1, pp. 22–31, 2016.

[16] A. Gyorgy, J. I. Jiménez, J. Yazbek, H.-H. Huang, H. Chung, R. Weiss, and D. Del Vecchio, "Isocost lines describe the cellular economy of genetic circuits," *Biophysical journal*, vol. 109, no. 3, pp. 639–646, 2015.

[17] J. Kim, A. P. Darlington, S. Muñoz-Montero, R. Montenegro, P. Dalby, N. Herrera-Martín, A. Banks, S. Prakash, K. Polizzi, D. Bates, and J. Jiménez, "Gene expression depends on the interplay among growth, resource biogenesis, and nutrient quality," *ACS Synth Biol*, vol. 14, no. 6, pp. 2012–2029, 2025.

[18] Y. Guan, X. Chen, B. Shao, X. Ji, Y. Xiang, G. Jiang, L. Xu, Z. Lin, Q. Ouyang, and C. Lou, "Mitigating host burden of genetic circuits by engineering autonegatively regulated parts and improving functional prediction," *ACS Synthetic Biology*, vol. 11, no. 7, pp. 2361–2371, 2022.
